# Protocol for *in vivo* assessment of glucose control and insulin secretion and sensitivity in the pig

**DOI:** 10.1016/j.xpro.2025.103774

**Published:** 2025-04-16

**Authors:** Yasmin Eckstein, Barbara Kessler, Arne Hinrichs, Istvan Novak, Anne von Thaden, Timo Lorenzen, Birgit Rathkolb, Armin Scholz, Andreas Blutke, Sietse-Jan Koopmans, Martin Hrabĕ de Angelis, Berit Østergaard Christoffersen, Eckhard Wolf, Simone Renner

**Affiliations:** 1 Chair for Molecular Animal Breeding and Biotechnology, Gene Center and Department of Veterinary Sciences, LMU Munich, 81377 Munich, Germany; 2 Center for Innovative Medical Models (CiMM), LMU Munich, 85764 Oberschleißheim, Germany; 3 German Center for Neurodegenerative Diseases (DZNE), LMU Munich, 81377 Munich, Germany; 4 Institute of Veterinary Pathology at the Center for Clinical Veterinary Medicine, LMU Munich, 80539 Munich, Germany; 5 German Mouse Clinic (GMC) and Genome Analysis Center (GAC), Institute of Experimental Genetics, Helmholtz Munich, 85764 Neuherberg, Germany; 6 Livestock Center Oberschleissheim, LMU Munich, 85764 Oberschleißheim, Germany; 7 Wageningen Livestock Research, Wageningen University & Research, Wageningen, the Netherlands; 8 Novo Nordisk A/S, Maaloev, Denmark; 9 German Center for Diabetes Research (DZD), 85764 Neuherberg, Germany; 10 Interfaculty Center for Endocrine and Cardiovascular Disease Network Modelling and Clinical Transfer (ICONLMU), LMU Munich, 81377 Munich, Germany; 11 Chair of Experimental Genetics, TUM School of Life Sciences (SoLS), Technische Universität München, 85354 Freising, Germany

**Keywords:** Metabolism, Model Organisms, Biotechnology and bioengineering

## Abstract

The pig is a valuable animal model in diabetes research; however, standardized protocols are essential for evaluating *in vivo* metabolism. Here, we present a protocol for *in vivo* assessment of glucose control and insulin secretion and sensitivity in the pig. We describe steps for catheter implantation, testing of intravenous glucose tolerance, performance of hyperinsulinemic-euglycemic clamps (HECs) and hyperglycemic clamps (HGCs), and blood processing. We then detail procedures for analysis of plasma glucose, insulin, glucagon, and C-peptide concentrations as well as data analysis.

For complete details on the use and execution of this protocol, please refer to Renner et al.^1^ and Renner et al.^2^

## Highlights

• Phenotyping recommendations for metabolic research in the pig
• Refined catheter insertion techniques for stress-free blood sampling
• Techniques for intravenous glucose tolerance test and glucose clamps
• Recommendations for sample processing and relevant hormone assays

## Abstract (editor highlights)

Publisher’s note: Undertaking any experimental protocol requires adherence to local institutional guidelines for laboratory safety and ethics.

## Graphical abstract

**Figure:**
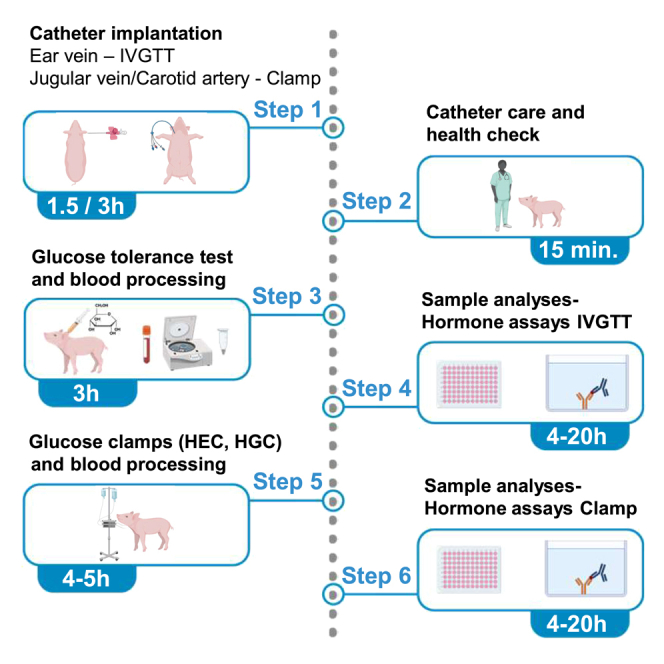

## Before you begin

### General considerations

The worldwide prevalence of diabetes mellitus is constantly rising and currently around 537 million people are affected.^3^ Diabetes mellitus is associated with severe micro- and macrovascular complications^4^ that in numerous cases are fatal.^5^ However, triggers of diabetes complications and their pathomechanisms are still not fully understood. So far, there is no cure for diabetes mellitus despite numerous treatment options. The pig as a model organism is of great value due to numerous anatomical and physiological similarities to humans, favorable reproductive characteristics, and the potential for genetic modification.

In this protocol, we provide for the first time step-by-step phenotyping procedures generally relevant for metabolism research and more specifically for diabetes and obesity research. In detail, we show the execution of an intravenous glucose tolerance test (IVGTT) and glucose clamps in the pig, the gold standard methods for the evaluation of insulin secretion and sensitivity. As GTTs and glucose clamps require frequent blood sampling, an appropriate vascular access is mandatory. Therefore, we additionally describe a refined minimal invasive technique for the placement of a central venous catheter into the auricular vein to perform an IVGTT as well as the placement of central venous and arterial catheters into the jugular vein and carotid artery for the performance of a glucose clamp. Both techniques are suitable for domestic pigs and minipigs of all age groups as well as for short term and long term use which is e.g., not the case for ultrasound-guided catheter placement techniques. They also allow repeated painless and stress-free blood sampling in unrestrained animals.

Before you start your experiment, several preliminary considerations are required dependent on your research question:

First, you need to decide which pig breed you would like to use for your experiment. Dependent on the location of your institution there are several domestic and minipig breeds available (Table 1). You also need to consider that dependent on the age group you would like to examine you have to handle pigs with a body weight of 200–300 kg for domestic pig vs. 30–100 kg for minipig breeds in adulthood. Furthermore, breed-related differences in body composition should be considered dependent on the research question to be answered (Table 2). In comparison to minipigs, domestic pig breeds have more than one auricular vein per ear that can be used for the placement of a central venous catheter allowing for easy catheter replacement in long-term studies. Also, blood should always be taken at the same time of day, as many hormones follow a circadian rhythm.^6^^,^^7^ Further, you should decide on the frequency and intervals of blood sampling required for your study to determine the implantation duration of the catheters accordingly. The implantation duration depends e.g., on the pig breed as well as on the implantation method and catheter care (see step 1-53). Finally, dependent on the age group, size and expected growth of your study animals select the appropriate catheter size and length.

**Table 1:** Selected pig breeds for biomedical research

Breed	Size (BW adult animal)	Reference
Göttingen	Minipig (35–45 kg)	Renner et al.^1^ Lutzhoft et al.^8^ Ellegaard GM^9^
Iberian	Domestic pig (100 kg)	Rodriguez-Lopez et al.^10^ Rodriguez et al.^11^
Landrace	Domestic pig (200–300 kg)	Rodriguez-Lopez et al.^10^; te Pas et al.^12^ Renner et al.^2^^,^^13^
Libechov	Minipig (70–90 kg)	Schubert et al.^14^
Mangalica	Domestic pig (100–125 kg)	Hallowell et al.^15^
Ossabaw	Minipig (30–70 kg)	Badin et al.^16^ Sham et al.^17^
Swabian Hall	Domestic pig (200–300 kg)	Renner et al.^2^
Yorkshire	Domestic pig (200–300 kg)	te Pas et al.^12^
Yucatan	Minipig (70–85 kg)	Ochoa et al.^18^ Jurrissen et al.^19^

**Table 2:** Body composition of selected pig breeds determined by dual-energy X-ray absorptiometry (DXA - GE Lunar DPX-IQ, adult normal) at the Livestock Center of LMU Munich

	DE	DL	DE-DL	IB	LB	Pi	SH	GM
N	23	134	41	18	54	17	11	27
BMD [g/cm^2^]	1.146 ± 0.016	1.190 ± 0.011	1.154 ± 0.014	1.139 ± 0.016	1.145 ± 0.009	1.131 ± 0.017	1.158 ± 0.021	1.038 ± 0.03
Fat mass [kg]	15.01 ± 1.49	22.03 ± 0.99	15.78 ± 1.25	20.62 ± 1.44	30.16 ± 0.86	13.76 ± 1.54	21.79 ± 1.96	3.66 ± 1.01
Lean mass [kg]	70.34 ± 1.74	72.93 ± 1.16	73.47 ± 1.46	41.69 ± 1.68	54.58 ± 1.01	76.48 ± 1.79	70.24 ± 2.28	29.66 ± 3.05
BMC [kg]	2.74 ± 0.11	3.11 ± 0.07	2.86 ± 0.09	1.96 ± 0.11	2.57 ± 0.06	2.73 ± 0.11	2.99 ± 0.14	0.93 ± 0.08
Total tissue [kg]	88.08 ± 2.87	98.07 ± 1.92	92.11 ± 2.41	64.27 ± 2.78	87.31 ± 1.66	92.97 ± 2.97	95.02 ± 3.78	34.26 ± 0.40
Fat [%]	16.21 ± 1.10	21.69 ± 0.74	16.34 ± 0.93	32.23 ± 1.06	34.66 ± 0.64	14.21 ± 1.14	22.55 ± 1.14	12.49 ± 1.32
Age [days]	187 ± 4.9	192 ± 3.3	201 ± 4.1	188 ± 4.7	200 ± 2.8	197 ± 5.1	185 ± 6.4	470.1 ± 20.3

BMC = bone mineral content; BMD = bone mineral density; DE = German Edelschwein; DL = German Landrace; DE_DL = F1 of DE und DL; IB = Iberian pig (Cerdo Iberico); LB = Large Black; Pi = Pietrain; SH = Swabian Hall pig; GM = Göttingen Minipig; all animals followed a restrictive feeding regime; under ad libitum feeding conditions minipig breeds are more prone to an obese phenotype. Data presented as LSMEANS ± SE; model fixed effects: breed and sex.

### Institutional permissions

All animal experiments were performed according to the German Animal Welfare Act with permission of the responsible animal welfare authority. Pigs can be group-housed except for the period when they are equipped with a central venous catheter since it is very likely that companion pigs with direct contact will remove the central venous catheter from the equipped animal. Water is available ad libitum and the pigs are fed a commercial diet unless the experiment requires otherwise. Enrichment, e.g., balls, teething chains, alfalfa sticks, is available to the animals at all times.

Make sure that you have obtained all necessary permissions to carry out the animal experiment.

### Handling of the pigs

**Timing: 2 weeks (time period may vary dependent on the pig breed and the individual animal)**

1. Familiarize the pigs with the people who will carry out the test and get them used to be touched at their ears/neck: e.g., handfeed the pigs while touching the pig's ear/neck.

***Note:*** The better the animals are used to the processes beforehand the less stressful it is for the animals during the experiment and the more reliable data one will get.

### Single housing of the pigs

**Timing:2–3days**

2. Place the pigs in a single pen with enough enrichment as well as visual and olfactory contact to their conspecifics two to three days (dependent on the pig breed and the individual animal) prior to catheter placement.

***Note:*** This allows the animals to familiarize with the new environment and be more relaxed during the experiment.

***Note:*** Make sure that the training also takes place when the animals are single housed.

### Sterilization of swabs and instruments

**Timing: 1 day**

3. Sterilize swabs and surgical instruments at the latest the day before catheter placement.

***Note:*** The use of sterilized materials is mandatory to prevent infections.

### Fasting time before the surgery

**Timing: 12 h**

4. The pigs must be fasted for the catheter placement. Feed them for the last time in the afternoon/evening the day before surgery.

**CRITICAL:** Insufficiently fasted animals can vomit during induction of anesthesia, develop an aspiration pneumonia and in the worst case suffocate during anesthesia.

### Preparation of heparinized saline

**Timing: 5 min**

5. On the day of catheter placement prepare heparinized saline:
  a. Remove two milliliters of saline from a 500 mL bottle.
  b. Add two milliliters of heparin (25,000 I.U. per mL) into the bottle with 498 mL of saline (100 I.U. per mL saline).
  c. Shake gently and keep it at room temperature (approximately 20°C–23°C).

## Key resources table

**Table:** 

REAGENT or RESOURCE	SOURCE	IDENTIFIER
**Chemicals, peptides, and recombinant proteins**		

Azaporc (40 mg/mL)	Serumwerk Bernburg AG	3187
Braunol, 1,000 mL	B. Braun SE	9322531
Glucosteril 50% solution for infusion, 500 mL	Fresenius SE & Co. KGaA	2262091
Heparin-Natrium Braun (25,000 IE/5 mL)	B. Braun SE	2147217
Insuman rapid insulin (100 IU/mL injection solutions)	Sanofi	PZN 01483785
Isofluran CP (1 mL/mL)	CP-Pharma Handelsgesellschaft mbH	1214
Isotonic saline 0.9%, 500 mL	B. Braun SE	3200950
Kodan tincture forte uncolored	Schülke & Mayr GmbH	104005
Metacam (20 mg/mL)	Boehringer Ingelheim Vetmedica GmbH	59243133
Novaminsulfon (500 mg/mL)	Bela-Pharm GmbH & Co. KG	1687111
TauroLock Hep500	TauroPharm GmbH	TP-02-5
Ursotamin (100 mg/mL)	Serumwerk Bernburg AG	3169
Xylazine (20 mg/mL)	Serumwerk Bernburg AG	3192

**Critical commercial assays**		

Merck porcine insulin RIA	Merck KGaA	PI-12K
Mercodia glucagon ELISA	Mercodia AB	10-1281-01
Mercodia porcine C-peptide ELISA	Mercodia AB	10-1256-01
Mercodia porcine insulin ELISA	Mercodia AB	10-1200-01

**Experimental models: Organisms/strains**		

Domestic pig (German Landrace mix)	Own breeding	Male and female, 3–4 months, 6–7 months
Domestic pig (German Landrace mix)	Own breeding	Female, pregnant, non-pregnant, 1 year
Minipig (Auckland Island)	Own breeding^20^	Male and female, 1 year

**Software and algorithms**		

GraphPad Prism 10	GraphPad	NA
SAS 8.2	SAS	NA
BioRender	BioRender	NA

**Other**		

15 mm Compact anesthesia system with 1-L bag, Luer angle, and additional tube, ≥ 2 m	Intersurgical GmbH	2164000
Adhesive spray	reboVet Veterinär-Fachgroßhandel GmbH & Co. KG	152408
Agani hypodermic needle 21G, 0.8 × 16 mm	Terumo Europe NV	AN∗2116R1
Anesthesia mask	Midmark Corporation	93815028
Careflow central venous catheter kit Seldinger technique 2.5 F	Merit Medical	681614
Careflow central venous catheter kit Seldinger technique 3 F	Merit Medical	681643
CNC-5H 3-5Fr Hydrocoat catheter. 40–80 cm length, round tip with 2 fixed vessel suture retention beads and pre-attached Luer stub (position of retention beads variable)	Access Technologies	CNC-5H
Centrifuge 5804 R	Eppendorf SE	5805000010
Clear-Guard ventilation filter	Intersurgical GmbH	1544000
Cryo rack for 81 tubes	Greiner Bio-One GmbH	802225
Digital timer	Carl Roth GmbH	A802.1
Discofix three-way valve	B. Braun SE	4095111
Discofix three-way stopcock plus extension line	B. Braun SE	16502C
Disposable razor	Wilkinson Sword GmbH	W302338200
Dräger isoflurane vapor 2000	Drägerwerk AG & Co. KGaA	N/A
Ear catheter secure device	Thorsted's Maskinværksted	N/A
Eppendorf Safe-Lock tubes 1.5 mL	Eppendorf SE	0030 123.328
epT.I.P.S. standard pipet tips blue	Eppendorf SE	0030000927
epT.I.P.S. standard pipet tips yellow	Eppendorf SE	0030000889
ES compresses 10 × 10 cm	Paul Hartmann AG	401835
ES compresses 5 × 5 cm	Paul Hartmann AG	401821
Fisherbrand Traceable timer	Fisher Scientific GmbH	11745863
Fixomull stretch 10 × 10 cm	BSN Medical GmbH	02037-00
FREESTYLE Lite test strips without coding	Abbott GmbH	EV111842
Glucometer Freestyle Freedom Lite without coding	Abbott GmbH	70915-70
IN-Plug Luer-Lock with injection cap	Fresenius Kabi Deutschland GmbH	8501502
Leukoplast 2.5 cm × 5 m	BSN medical GmbH	01532-00
LifeVet 8M monitor	Eickemeyer	321900
Mini-Spike	B. Braun SE	4550242
Multi-adapter Luer for S-Monovette	Sarstedt AG & Co. KG	14.1205
Neptune anesthesia ventilator	Medec Benelux NV	N/A
Omnifix Luer 10 mL, off-center	B. Braun SE	4616103V
Omnifix Luer 20 mL, off-center	B. Braun SE	4616205V
Omnifix Luer 3 mL	B. Braun SE	4616025V
Omnifix Luer 5 mL, off-center	B. Braun SE	4616057V
OP-Cover (90 × 120 cm)	Dispovet	12120
Original Perfusor line PVC, 75 cm, 0.9 × 1.9 mm	B. Braun SE	8722870N
Original Perfusor line PVC, 50 cm, 1.5 × 2.7 mm	B. Braun SE	8255172
Original Perfusor syringe, 50 mL	B. Braun SE	8728844F-06
Perfusor space	B. Braun SE	8713030
Prolene 2/0, 75 cm, needle 3/8 circle, reverse cutting	Johnson & Johnson Medical GmbH	8666H
S-Monovette citrate 1:10 4.3 mL	Sarstedt AG & Co. KG	04.1922
S-Monovette citrate 1:10 8.2 mL	Sarstedt AG & Co. KG	01.1606.001
S-Monovette hematology/EDTA K 7.5 mL	Sarstedt AG & Co. KG	01.1605.001
S-Monovette hematology/EDTA K 2.7 mL	Sarstedt AG & Co. KG	05.1167
S-Monovette lithium-heparin 4.5 mL	Sarstedt AG & Co. KG	05.1106
S-Monovette lithium-heparin 7.5 mL	Sarstedt AG & Co. KG	01.1604
S-Monovette serum 4.5 mL	Sarstedt AG & Co. KG	05.1104
S-Monovette serum 7.5 mL	Sarstedt AG & Co. KG	01.1601
Soft sponge	N/A	N/A
Softaskin, 1.000 mL	B. Braun SE	180217
Spark multimode microplate reader	Tecan Trading AG	N/A
Spherasorb breathing lime	Intersurgical GmbH	2175000
Spiral extension set (ProSet), 300 cm	B. Braun SE	4092945
Steel pins W: 1.6 mm, L: 14 mm, 16 mm, 18 mm, 20 mm with ball 5 mm	Crazy Factory	N/A
tesa fabric tape 5 cm	Tesa SE	4651
Three-way valve benches (3- to 5-fold)	B. Braun SE	16605C, 16609C
Tunneling device	Custom-made	N/A
Vasco sensitive natural white latex surgical gloves, size 6.5	B. Braun SE	6081010
Vasco sensitive natural white latex surgical gloves, size 7.5	B. Braun SE	6081037
VasoVet intravenous catheter, 22G, 0.9 × 25 mm, blue	B. Braun SE	4269102
VasoVet intravenous catheter, 22G, 0.9 × 25 mm, pink	B. Braun SE	4269219
VasoVet intravenous catheter, 24G, 0.7 × 19 mm, yellow	B. Braun SE	4269075
Vicryl 2/0, 70 cm, reverse cutting	Johnson & Johnson Medical GmbH	V686H
Vortex-Genie 2	Scientific Industries, Inc.	SI-0236

## Step-by-step method details

### Catheter implantation (ear vein)—Day 1

**Timing: 1–1.5 h**

**Figure 1:**
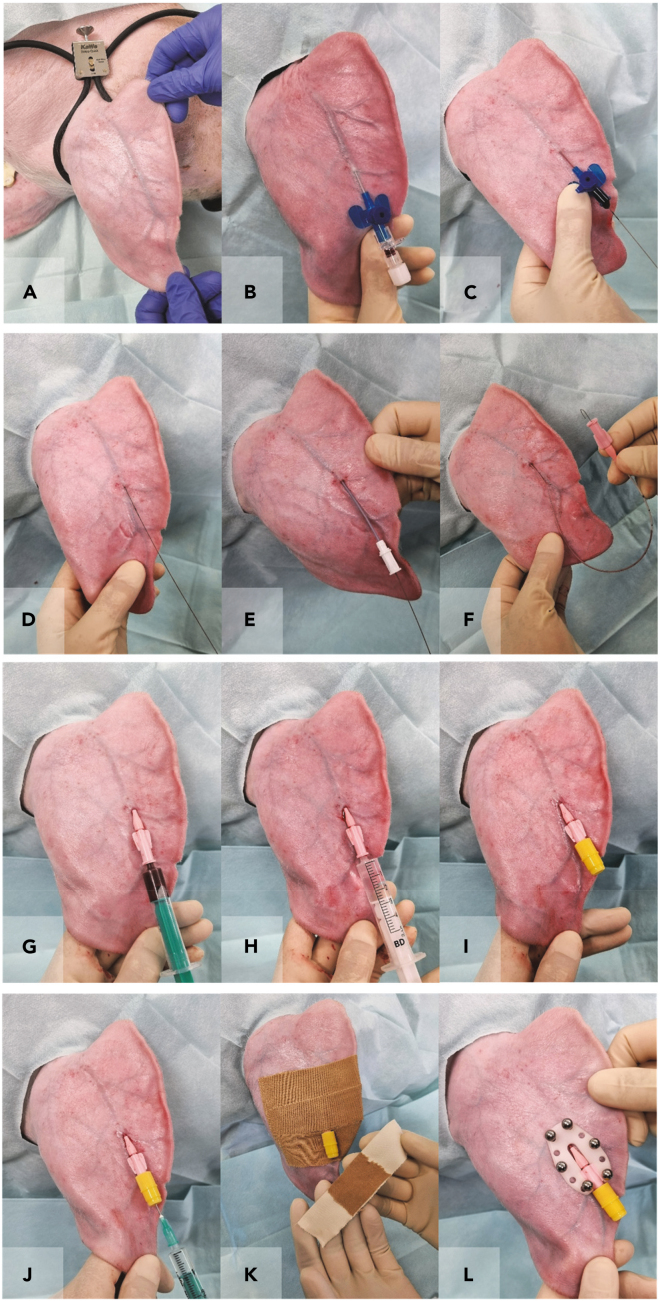
Over the wire central venous catheter placement in an auricular vein Disinfect the pig’s ear and place a tourniquet at the ear base (A). Insert a venous catheter (B), remove the cannula and insert the wire through the venous catheter into the auricular vein (C). Remove the venous catheter (D) and enlarge the puncture site using the dilatator (E). Remove the dilator, insert the central venous catheter over the wire (F) and remove the wire thereafter. Draw blood from the central venous catheter (G) and flush it with heparinized saline (H). Seal the catheter with a plug (I) and insert catheter lock solution through the membrane of the plug (J). Fixate the catheter with a single suture at the designated deepening and secure it with tape (K) or with a holding device and steel pins (L). See also Figures S1–S3.

A central venous catheter is placed into a porcine ear vein (Figures 1 and S1–S3; Methods video S1).

***Note:*** Perform the catheter implantation with at least one, preferably two assistants. One person is assisting during the surgery, the second person is responsible for monitoring the anesthesia and the sterile handing of equipment.

***Note:*** The animals should be fasted overnight (approximately 12–18 h).

1. Anesthetize the pig:
  a. Induce anesthesia by intramuscular injection using e.g., a combination of Ketamine (20 mg/kg BW) and Azaperone (2 mg/kg BW).
  b. Maintain anesthesia using e.g., Isoflurane (1–1.5%).
  c. Provide the pig with adequate analgesia, e.g., Metamizole (15–50 mg/kg BW i.v.).

**CRITICAL:** Warm the pig during anesthesia by using e.g., a heating mat to prevent an excessive drop of the pig’s body temperature and therefore reduce post-surgical discomfort, muscle tremor as well as maintain plasma clearance of anesthetics. Uncontrolled movements during the wake-up phase also increase the risk of damage to the catheter.

2. Prepare the pig's ear for catheter insertion:
  a. Wash the ear to be used thoroughly with soap using a soft sponge.
  b. Rinse the ear with water and dry it.
  c. Carefully shave the front and back of the ear.

**CRITICAL:** Proceed with caution and use a soft sponge to avoid possible skin irritation and thus prevent potential sources of infection. Skin irritations due to too ambitious shaving of the very soft skin at the ear can lead to pruritus and can cause damage of the catheter due to continuous scratching by the animal.

3. Transfer the pig to an operating table and connect it to a suitable anesthesia monitoring system.
4. Disinfect the ear thoroughly:
  a. Start the disinfection with iodine solution (7.5%), always wiping in one direction, away from the puncture site, and using a new swab with each disinfection cycle.
  b. Allow the iodine solution to dry and repeat the process.
  c. Finally, spray the ear with a 2-propanol based disinfectant and leave it to dry.

***Note:*** Thorough disinfection and sterile hygienic working conditions are required to avoid possible infections and enable to avoid the use of antibiotics.

5. Place a tourniquet around the base of the ear and cover the area around the ear with a sterile drape (Figure 1A).
6. Have the blood flow in the ear veins blocked by gently tightening the tourniquet and place a venous catheter (e.g., VasoVet, B.Braun) in the blocked ear vein with sterile gloves (Figure 1B).

***Note:*** Choose 24 gauge (24 G) venous catheters for piglets (∼up to 20 kg BW), 22 G catheters for adolescent pigs (∼up to 60 kg BW) and 20 G catheters for young adult and adult pigs.

***Note:*** If possible, choose a vein with a straight course for easy advancement of the catheter as well as an adequate distance to the ear margin so that the catheter does not protrude beyond the edge of the ear and can be secured safely on the ear.

7. Insert the wire into the ear vein (Figures 1C and 1D) **–** potential challenge, see troubleshooting guidelines:
  a. Remove the inner metal cannula.
  b. Insert the wire into the remaining plastic-tube of the catheter up to approximately two-thirds of its length.
  c. Remove the plastic-tube from the vein over the wire.
  d. Wipe away any blood from the wire with a sterile swab moistened with heparinized saline.

**CRITICAL:** Be careful not to pull out the wire when removing the venous catheter or when cleaning the wire.

8. Slide the dilator over the wire and enlarge the puncture site in the skin. Withdraw the dilator. (Figure 1E).

***Note:*** Wipe away any blood from the wire with a sterile swab moistened with heparinized saline.

***Note:*** It is important to enlarge the puncture site, as the central venous catheter is very soft and otherwise cannot be pushed through the skin into the vein.

9. Insert the central venous catheter into the ear vein (Figure 1F) potential challenge, see troubleshooting guidelines:
  a. Slide the central venous catheter over the wire, but not yet into the ear vein.
  b. Push the wire back and at the same time push the catheter forward right at the insertion site until you can take hold of the wire at the end of the catheter with your fingers.
  c. Ask your assistant to hold the wire in place while at the same time you push the catheter over the wire into the vein.

**Figure 2:**
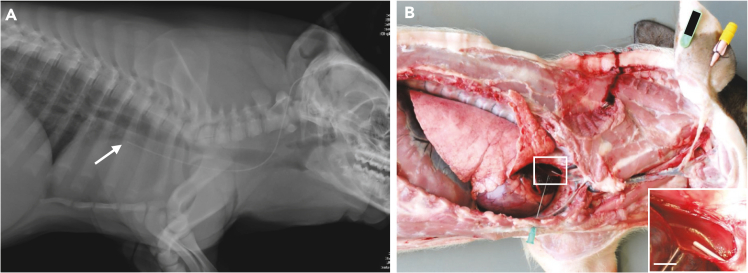
Course and position of the ear vein catheter Latero-lateral chest radiograph (A) and necropsy situs (B) displaying the position of the ear catheter tip (red arrow). (B) Lateral aspect of the opened thorax. The anterior caval vein is fenestrated. The tip of the catheter (arrow) is positioned in front of the right atrium (rectangle). Inset: Detail enlargement of the catheter tip, scale bar 1 cm.

**CRITICAL:** Measure the distance from the insertion site to the first rib for an estimate of the distance the catheter can be inserted into the vein (relevant for pigs with a BW up to ∼40 kg). The catheter cannot be inserted further than to the base of the heart and should never be advanced into the atrium. Verify the distance by x-ray at least once for each age group (Figure 2).

10. When the central venous catheter is completely inserted into the ear vein, carefully pull the wire out of the vein.
11. Immediately draw some blood from the central venous catheter with a 3-mL syringe to check the correct position in the vein. Then flush 5 mL of heparinized saline into the catheter to prevent blood clotting in the catheter (Figures 1G and 1H).

***Optional:*** Take a blood sample for hematology and clinical chemical evaluation as a baseline to be compared with samples from test days (IVGTT or clamp) to control for subclinical inflammation related to the catheter that could interfere with your research question.

12. Place a catheter plug with diaphragm at the end of the central venous catheter and insert 1 mL of catheter lock solution, e.g., Tauro Lockf into the catheter using a short 21G cannula (21 G, 0.80 × 16 mm) (Figures 1I and 1J).
13. Clean the central venous catheter and the area around the ear with sterile moistened swabs.

***Note:*** Remove all blood from the ear as this is a source of infection.

14. Secure the central venous catheter to the ear by using sutures or steel pins.
  a. Suture (Figures 1K and S2):
    i. Fixate the catheter to the ear with one single suture piercing all layers of the ear.
    ii. Cover the puncture site with a small piece of swab soaked in iodine solution (7.5%).
    iii. Secure the central venous catheter to the ear with tape.
    iv. Use a special adhesive to cover the catheter plug.
      ***Note:*** Use a monofilament, non-absorbable suture (e.g., Prolene 2/0) with a circular cutting needle.
      **CRITICAL:** Do not use too much tape, otherwise the pig will become irritated and will shake its head frequently, which can lead to an earlier loss of the catheter.
  b. Steel pins (Figures 1L and S3):
    i. Use pliers to fixate the catheter into the holding device.
    ii. Keep the catheter with the holding device in position, so that it cannot bend.
    iii. Check the six pin openings for underlying small vessels.
    iv. Insert a cannula of a suitable size (e.g., 14 G, 21 × 80 mm) at each selected opening.
    v. Remove it and insert the steel pin (thickness 1.6 mm) afterward.
    vi. Place at least four pins (better five to six pins for domestic pig breeds) to secure the catheter safely to the ear starting with the outer openings.
    vii. Use thread glue to permanently seal the balls of the steel pins.
      ***Note:*** Choose the length of the pins according to the thickness of the ear, e.g., 14, 16, 18 or 20 mm. There should be ∼2–3 mm distance from the steel ball to the ear, keep in mind that initially there will be some swelling and therefore do not choose too short pins.
      **CRITICAL:** Make sure that the thread glue does not contact the pig’s skin as it is an irritant.
      ***Note:*** Depending on the indwelling time of the central venous catheter in the pig’s ear, choose suture (up to three to four weeks) or steel pins (from four weeks) as the preferred fixation method.
      **CRITICAL:** Make sure that you do not puncture an underlying vessel when using suture or steel pins, as this is a significant source of infection.
15. End the anesthesia and remove the monitoring system from the pig. Transfer the pig to a suitable wake-up pen and warm it with a red-light lamp during the wake-up phase.

***Note:*** Provide the pig with non-steroidal antiphlogistics (NSAIDs), e.g., Meloxicam (0.4 mg/kg BW) for two days following catheter implantation with suture and for three days following catheter implantation with steel pins.

**CRITICAL:** Make sure the pen is always clean and dry (!) to avoid catheter infection and loss. The pen must not have any edges that the pig can use to pull out the catheter.

Document S1. Figures S1–S6

Methods video S1. Implantation of a catheter into the auricular vein (steps 6–15)

Methods video S2. Implantation of a catheter into the internal jugular vein and carotid artery (steps 26–33)

### Catheter care and health check—Days 2 and 3

**Timing: 5–15 min per day**

The catheter is flushed with heparinized saline and treated with a lock solution. A brief examination focusing on the ear with the catheter and internal body temperature measurement are performed.

16. Check the pig’s general health and the hygiene in its pen.

***Note:*** The pen should be dry-cleaned several times a day to prevent possible infection.

17. Examine the ear with the catheter more closely. Pay particular attention to increased heat, redness, swelling or other signs of inflammation - potential challenge, see troubleshooting guidelines.

***Note:*** If there are signs of inflammation, treat the pig with a suitable analgesic, e.g., Meloxicam (0.4 mg/kg BW), an antipyretic and/or an antibiotic if needed. Remove the catheter if necessary.

18. Check the position of the catheter and the catheter plug. Check the tape securing the catheter.

***Note:*** Remove tape that is no longer attached to the ear and replace it.

19. Flush the central venous catheter, potential challenge, see troubleshooting guidelines:
  a. If necessary, remove the special adhesive.
  b. Clean and disinfect the catheter plug using a 2-propanol based disinfectant and swabs.
  c. Remove the Tauro Lock from the catheter and thereafter flush the catheter with 5 mL of heparinized saline through the diaphragm of the catheter plug using a short 21 G cannula (0.80 × 16 mm).
  d. Insert 1 mL of catheter lock solutions, e.g., Tauro Lock into the catheter using a short 21 G cannula (0.80 × 16 mm).
  e. Cover the catheter plug with a special adhesive if necessary.
  f. If the catheter is attached with pins, check that the balls of the pins are firmly in place. Clean the holding device, the pins as well as the skin underneath the holding device with a slightly moistened swab and/or moistened cotton swabs to prevent skin irritation and infection.

***Note:*** Use food to distract the pig and get better access to the catheter ear.

***Note:*** Change the catheter plug three times a week, or more often if necessary. Flush the catheter two to three times per week.

20. Measure the pig’s internal body temperature.

**Pause Point:** Continue with the glucose tolerance test not earlier than the day after next. You can also postpone the IVGTT to a later day.

### Catheter implantation (jugular vein and carotid artery)—Day 1

**Timing: 2–3 h**

A central catheter is placed into the internal jugular vein and carotid artery (Figure 5; Methods videos S2 and S3).

***Note:*** Perform the catheter implantation with at least one, preferably two assistants. One person assists during the surgery, the second person is responsible for monitoring the anesthesia and the sterile handing of equipment.

***Note:*** The animals should be fasted overnight (approximately 12–16 h).

21. Anesthetize the pig:
  a. Induce anesthesia by intramuscular injection using e.g., a combination of Ketamine (20 mg/kg BW) and Azaperone (2 mg/kg BW).
  b. Maintain anesthesia using e.g., Isoflurane (1–1.5%).
  c. Provide the pig with adequate analgesia, e.g., Metamizole (50 mg/kg BW i.v.).

**CRITICAL:** Warm the pig during anesthesia by using e.g., a heating mat to prevent an excessive drop of the pig’s body temperature and therefore reduce post-surgical discomfort, muscle tremor as well as maintain plasma clearance of anesthetics. Uncontrolled movements during the wake-up phase also increase the risk of damage to the catheter.

22. Prepare the pig’s neck for catheter insertion:
  a. Wash the entire neck (dorsal and ventral site) of the pig thoroughly with soap using a soft sponge.
  b. Rinse the neck with water and dry it with a towel or wipes.
  c. Carefully shave the entire neck.

**CRITICAL:** Proceed with caution and use a soft sponge to avoid possible skin irritation and thus prevent potential sources of infection.

23. Transfer the pig to an operating table:
  a. Place the pig in exact dorsal recumbency.
  b. Secure the front legs with soft ropes so that they point towards the hind legs to get the ventral skin of the neck under tension.
  c. Connect the animal to a suitable anesthesia monitoring system.
24. Disinfect the operating field - right side of the pig's neck around the jugular groove:
  a. Start the disinfection with iodine solution (7.5%), always wiping in one direction, away from the surgical access point, and using a new swab with each disinfection cycle.
  b. Allow the iodine solution to dry and repeat the process.
  c. Finally, spray the operating field with a 2-propanol based disinfectant and leave it to dry.

***Note:*** Thorough disinfection and sterile hygienic working conditions are required to avoid possible infections and enable to avoid the use of antibiotics.

25. Cover the surgical field either with a self-adhesive cover sheet or use skin adhesive spray to fix the cover sheet.
26. Make a skin incision about five centimeters long in the sulcus medial to the jugular groove (medial to the sternocephalic muscle, mid of the neck) (Figure 3A).

**Figure 3:**
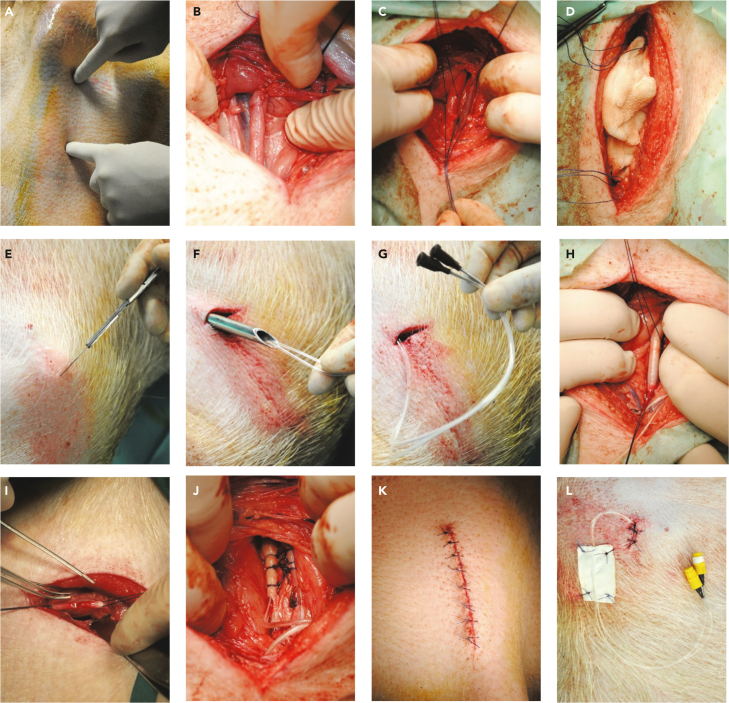
Jugular vein and carotid artery catheter placement with subcutaneous tunneling Landmarks – sulcus medial to the jugular groove - for the cutaneous access to the internal jugular vein and carotid artery (A); Blunt dissection to the vessels and vagal nerve (B); Placing of holding sutures to secure vein and artery (C); Coverage of vein and artery with a swab soaked with heparinized saline to proceed with tunneling (D); Skin incision at the back of the pig for easy exit of the tunneling device (E); Insertion of both catheters through the tunneling device (F); Pulling of the tunneling device (G); Pre- positioning of the artery using the holding sutures (H); Incision of artery and insertion of the catheter into the artery (I); Securement of catheter into the artery with sutures (J); Closure of the surgery site (K); Securement of the catheters to the skin (L) See also Figure S4.
27. Continue with blunt preparation through the muscle layer and muscle fascia.

**CRITICAL:** Work slowly and carefully to avoid damaging blood vessels.

***Note:*** The use of an electric coagulation system is beneficial but manual coagulation of small bleedings that might occur is also possible.

28. Expose the bundle of carotid artery, internal jugular vein and vagal nerve and carefully dissect of connective tissue from the carotid artery and the internal jugular vein over a length of approximately five centimeters (Figure 3B).

***Note:*** Prepare the tissue between the structures bluntly wherever possible.

29. Apply two holding sutures, one at each side of the exposed artery and vein (Figure 3C).
30. Secure the operating field (Figure 3D):
  a. Secure the two holding sutures with surgical clamps.
  b. Cover the surgical site with saline-soaked swabs.
  c. Place the pig in lateral recumbency (right side up).
31. Place the tunneling device at the incision site and advance it subcutaneously along the right side of the neck up to the back.
32. Have your assistant disinfect the exit point and make a skin incision with a length of one to two centimeters to facilitate the exit of the tunneling device (Figure 3E).
33. Guide the two catheters through the tunneling device (Figure 3F) and thereafter remove the tunneling device (Figure 3G).
34. Add a cap to both Luer Lock adapters to lock the catheters and flush the catheters with saline.

***Note:*** Mark the venous and arterial catheter at the Luer Lock site accordingly to recognize them at any time.

35. Place the pig in dorsal recumbency again.
36. Remove the soaked swabs and let your assistant gently lift the artery to keep it in your field of vision and interrupt blood flow (Figure 3H).

***Note:*** Ligate possible smaller vessels that branch off from the artery.

**CRITICAL:** Make sure to remove all connective tissue especially at the arteriotomy site as it can cover the opening and therefore complicate to advance the catheter into the artery.

37. Tighten the holding sutures to block the blood flow. Cut a small hole (2–3 mm) into the artery with pointed scissors and remove any leaking blood with saline-soaked swabs (Figure 3I).

***Note:*** Do not cut the hole too large so that the artery does not tear.

***Note:*** You can directly control the blood flow in the artery towards the site of the arteriotomy (and thereby potential bleeding) by lifting or releasing the caudal holding suture.

38. Insert the catheter in caudal direction into the artery up to the second suture retention bead and place a crossing ligature cranial to the insertion site to prevent bleeding from the artery (Figure 3J).

**Figure 4:**
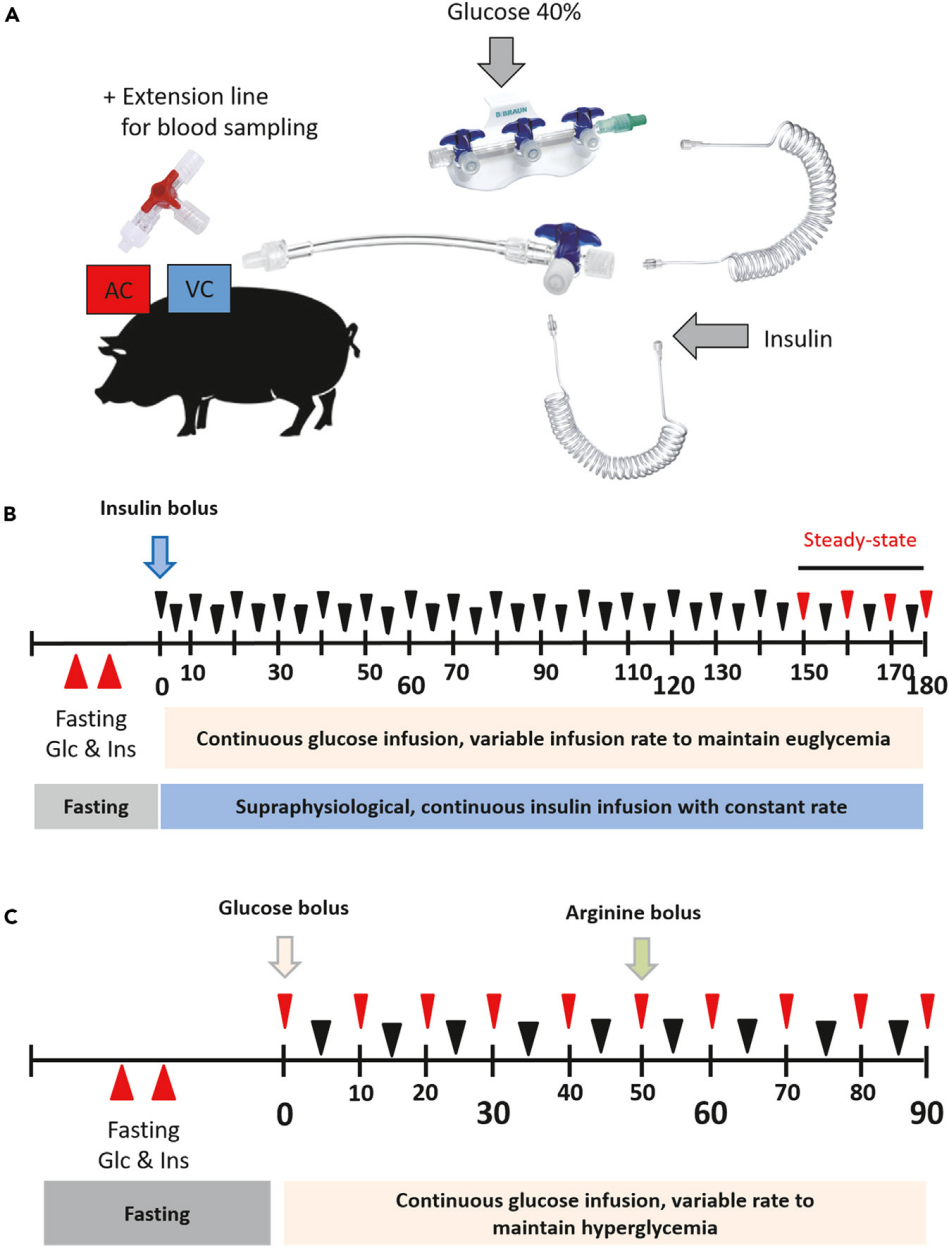
Schematic outline for performing a hyperinsulinemic-euglycemic and hyperglycemic clamp Outline of the infusion and blood sampling through venous and arterial catheter (A); Outline of a hyperinsulinemic-euglycemic clamp (HEC) (B); Outline of a hyperglycemic clamp (HGC) (C); AC: arterial catheter, VC: venous catheter, black triangles: blood sampling for blood glucose check, red triangles: blood sampling for blood glucose check and further analyses.

**CRITICAL:** Measure the distance from the skin incision site to the first rib for an estimate of the distance the catheter can be inserted into the artery. The catheter cannot be inserted further than to the base of the heart and should never be advanced into the atrium. Verify the distance by x-ray at least for each age group (Figure 4).

***Note:*** The catheter is equipped with two non-movable suture retention beads allowing the adjustment of the correct distance to be inserted into the artery. The suture retention beads have to be placed beforehand at the respective distance of the catheter one centimeter apart from each other.

39. Place a ligature between the two beads of the catheter as well as behind the second bead to hold the catheter in place and prevent bleeding (Figure 3J).

**CRITICAL:** Do not pull the knot too tight to ensure blood flow, but also not too loose so that the catheter cannot slip out of the artery.

40. Aspirate blood with a 3-mL syringe to proof the catheter`s patency and flush the arterial catheter with heparinized saline thereafter.

***Optional:*** Take a blood sample for hematology and clinical chemical evaluation as a baseline to be compared with samples from test days (IVGTT or clamp) to control for subclinical inflammation related to the catheter that could interfere with your research question.

41. Repeat steps 36 to 40 for the internal jugular vein.
42. Remove blot clots in case there are any, rinse the wound cavity with warm saline and dry everything with sterile swabs.
43. Suture the muscle layer using a continuous stitching and an absorbable suture material (e.g., vicryl).
44. Suture the skin using single stitches and a non-absorbable suture material (e.g., prolene) (Figure 3K).

***Note:*** You can also perform an intradermal suture, which has the advantage that no suture material is visible and can be an entry point for infection.

45. Flush the catheters:
  a. Check the patency of the catheters by aspirating blood again using a 3-mL syringe.
  b. Flush the arterial and venous catheter with heparinized saline.
  c. Inject catheter lock solution, e.g., Tauro Lock, through the diaphragm using a short 21 G cannula (0.80 × 16 mm) dependent on the volume of the catheters.

***Note:*** Dependent on the size of the pig and the length of the catheters you chose it might be necessary to connect an extension line to the catheters for easy access and to avoid tension on the catheters.

46. Attach the catheters to the skin (Figure 3L):
  a. Relocate the pig to lateral recumbency.
  b. Form a loop with the catheters and add a small piece of tape forming two flaps at the end of the loop.
  c. Secure the catheters with a single stitching to the skin.

***Note:*** The loop helps to keep away tension from the catheters.

47. Form a pouch using e.g., swabs and place the ends of the catheters into the pouch. Secure the pouch to the back of the pig with tape (Figure S4).
48. Mark the site where the catheter caps are placed with a pen so that you can re-open the pouch with a scalpel blade and/or scissors and have easy access for further catheter handling.
49. End the anesthesia and remove the monitoring system from the pig. Transfer the pig to a suitable wake-up pen and warm it with a red-light lamp during the wake-up phase.
  ***Note:*** Provide the pig with analgesics for three to five days after the surgery, e.g.
  a. Metamizole (15–50 mg/kg BW) i.v. injection every 6 h for the first 24 h and Meloxicam (0.4 mg/kg BW) intramuscular injection or oral administration every 24 h until day 5 after surgery.
  b. Buprenorphine i.v. injections (0.01–0.02 mg/kg BW) every 6 h for the first 24 h and Meloxicam (0.4 mg/kg BW) intramuscular injection or oral administration every 24 h until day 5 after surgery.
  c. Buprenorphine i.v. injection (0.05 mg/kg) at the end of surgery in combination with buprenorphine patches for long-term pain relief. Patch placement:
    i. Shave and clean the area on the thin skin behind the ears.
    ii. Disinfect the area with ethanol and allow it to dry completely.
    iii. Place the buprenorphine patch(es) on the clean, dry skin in a size and number giving rise to a transdermal dose of 5.25 μg/kg/time.
    iv. Secure the buprenorphine patches using a bandage of gauze and Tensoplast 7.5 cm.
    v. Remove the bandage after 5–7 days.
      **CRITICAL:** Make sure the pen is always clean and dry (!) to avoid catheter infection and loss. The pen must not have any edges that the pig can use to pull out the catheter.

### Catheter care and health check—Day 2 to day 6

**Timing: 5–15 min per day**

The catheters are flushed with heparinized saline and treated with a lock solution. A brief clinical examination and measurement of internal body temperature are performed.

50. Check the pig’s general health and the hygiene in its pen.

***Note:*** The pen should be dry-cleaned several times a day to prevent possible infection.

51. Examine the surgery site at the neck for any heat, redness, swelling or other signs of inflammation. Check the pig’s internal body temperature - potential challenge, see troubleshooting guidelines.

***Note:*** If there are signs of inflammation, treat the pig with a suitable analgesic, e.g., Meloxicam (0.4 mg/kg BW), an antipyretic and/or an antibiotic if needed. Remove the catheter if necessary.

52. Check the position of the catheter pouch and the tape securing it.
53. Flush the catheter(s) - potential challenge, see troubleshooting guideline**s**:
  a. Aspirate blood (at least the volume of the catheter and if applicable of the extension line) to remove any blood clots that might have formed and the catheter lock solution. This is especially important for the arterial catheter!
  b. Flush the arterial and venous catheter with 5 mL of heparinized saline.
  c. Insert catheter lock solutions, e.g., Tauro Lock, through the diaphragm of the catheter plug using a short 21 G cannula (0.80 × 16 mm).
  d. Put the catheters back into the catheter pouch and close the pouch opening with tape.

***Note:*** Repeat the catheter care and the health check every day.

**Pause Point:** Continue with the glucose clamps 6 days later. You can also postpone the clamps to a later day.

### Intravenous glucose tolerance test—Day 4

**Timing: 2.5–3 h**

An IVGTT is performed with glucose injection and blood sampling through the auricular central venous catheter.

***Note:*** The pig should be fasted for 12–18 hours before starting the IVGTT.

**CRITICAL:** The fasting interval should be the same range for each animal from the same study (± one hour).

54. Flush an extension line (50 cm, volume 2.1 mL) with heparinized saline and place a plug on the end of it.

***Note:*** For smaller pigs you can also choose extension lines with smaller volumes (e.g., 75 cm, volume 0.8 mL).

**CRITICAL:** Make sure that there is no air in the extension line as air in the bloodstream can trigger an air embolism.

55. Remove the special adhesive tape from the pig’s central venous catheter and remove the catheter plug.
56. Connect the extension line to the catheter and cover the catheter again with the special adhesive tape. Use tape to attach the extension line to the pig’s neck and back.

***Note:*** Make sure that the extension line is not in the direct field of view of the animal as this can be a distraction. You can achieve this by forming a small loop and attaching the extension line directly at the ear.

***Note:*** Make sure that there is no tension on the extension line even when the animal lowers its head. You can achieve this by forming a loop before attaching the extension line to the neck and back.

**CRITICAL:** Attach the extension line close to the pig's body in loops and make sure that the pig cannot hook itself anywhere in the pen with the extension line so that no tension is exerted on the catheter.

57. Check the patency of the catheter by flushing it with heparinized saline.

**CRITICAL:** Be careful not to pull on the extension line and thus on the catheter to avoid pulling the catheter out of the vein.

58. Collect the first blood sample (time point −10 min) from the pig:
  a. Remove the plug of the extension line and use a 3-mL syringe to aspirate catheter lock or flush solution and blood from the catheter (amount dependent on the volume of the extension line and the catheter but should be the same for each animal and sample).
  b. Connect the first blood tube to the extension line and fill it up to the marking.
  c. Flush the extension line using 5 mL of heparinized saline.
  d. Set the plug back to the end of the extension line and start the timer.

***Note:*** Bend the extension line every time before you remove the plug, a syringe or a blood tube and only undo the bend when new tool is attached. This prevents air from getting into the extension line and thus into the blood circulation.

***Optional:*** Measure the blood glucose level directly after collecting the blood sample with a plasma calibrated glucometer and record the value. For more accurate results measure in duplicate and calculate the average value of the two measurements. If the values differ too much (> 10–15 mg/dL), perform a third or fourth measurement.

59. Repeat step 58 after 10 min for time point 0. Reset the timer.
60. Inject the glucose intravenously as a bolus:
  a. Remove the plug from the extension line.
  b. Restart the timer at the beginning of glucose injection.
    ***Note:*** Record the time needed for glucose injection and keep the injection time as constant as possible within one animal group.
  c. Inject 1 mL 50% glucose solution/kg BW (corresponding to 0.5 g glucose/kg BW) through the extension line as a bolus and have an assistant write down the injection time.
  d. Flush the extension line with 10 mL saline and clean the Luer Lock adapter of the extension line with a moistened swab to remove possible glucose residues.
  e. Put the plug back on the extension line.
  f. Change your gloves.
61. Repeat step 58 for the following time points: 1; 3; 5; 7; 10; 15; 20; 30; 40; 50; 60 and 90 min.

**CRITICAL:** To keep timing of blood withdrawal as constant as possible define an exact time window, record deviations.

***Note:*** Dependent on your research question and potential outcome you can choose other time points or prolong/shorten the test.

62. Remove the tape and the extension line.
63. Flush the central venous catheter:
  a. Put a new catheter plug on the catheter.
  b. Flush 5 mL of heparinized saline via the diaphragm of the catheter plug into the catheter using a short 21 G cannula (0.80 × 16 mm).
  c. Insert 1 mL of catheter lock solution, e.g., Tauro Lock into the catheter using a short 21 G cannula.
64. Cover the catheter plug with a special adhesive (Figure 1K).
65. Catheter removal: If you no longer need the catheter, remove it from the pig's ear.
  a. Suture fixation:
    i. Remove the adhesive tape.
    ii. Carefully cut the suture with a scalpel blade or suture scissors.
    iii. Slowly pull the catheter out of the vein.
    iv. Stop any bleeding that may occur with a swab and pressure on the puncture site.
      ***Note:*** If the pig is well handled, no anesthetic is needed to remove the catheter.
  b. Steel pin fixation:
    i. Anaesthetize the pig by using suitable intravenous anesthetics e.g., a combination of Ketamine (17.5 mg/kg BW) and Xylazine (1 mg/kg BW) or Propofol (4 mg/kg/h; Propofol 2%, 20 mg/mL).
    ii. Cut the pins with pliers.
    iii. Slowly pull the catheter out of the vein.
    iv. Stop any bleeding that may occur with a swab and pressure on the puncture site.
    v. Transfer the pig to a suitable wake-up pen and warm it with a red-light lamp during the wake-up phase.
      ***Optional:*** Define prerequisites for the evaluability of the IVGTT, e.g., maximum permissible deviation of fasting time, glucose injection time, and timing of blood withdrawal.

### Hyperinsulinemic-euglycemic clamp—Day 7

**Timing: 4–5 h**

An HEC is performed with blood sampling through the arterial catheter as well as glucose and insulin infusion through the venous catheter (Figures 4A and 4B, S5).

***Note:*** The pig should be fasted for 12–18 h before starting the HEC.

**CRITICAL:** The fasting interval should be the same range for each animal from the same study (± one hour).

***Note:*** The length of infusion lines depends on your experimental setup but should be as short as possible so that changes of the infusion volume will reach the pig’s blood circulation quickly.

66. Prepare enough heparinized saline:
  a. Remove one milliliter of saline from a 500-mL bottle.
  b. Add one milliliter of heparin (25,000 I.U. per mL) into a 500-mL bottle of saline (50 I.U. per mL saline).
  c. Shake gently and keep it at room temperature (approximately 20°C–23°C).
67. Prepare a 10 I.U. per 12 mL 0.9% saline insulin solution using e.g., Insuman Rapid (40 IU Insulin per 1 mL).
  a. Preload a 50-mL perfusor syringe with 48.75 mL of 0.9% saline.
  b. Inject 1.25 mL of insulin into the perfusor syringe, i.e., 1 I.U. insulin / mL 0.9% saline.
  c. Shake the perfusor syringe.
  d. Connect it to a spiral extension set and clamp it into the perfusor.
68. Prepare perfusor syringes with 40% glucose solution, connect them to an extension line and clamp them into a corresponding number of perfusors.

***Note:*** At least two perfusors for glucose infusion are required for easy perfusor syringe changes; whether more than two are needed depends on the body weight and insulin sensitivity of the pigs.

***Note:*** Dependent on the body weight and insulin sensitivity of the pigs and availability of perfusors also a lower concentrated glucose solution (e.g., 20%) can be used.

69. Place the pig into a space-reduced pen or reduce its own pen by size.
70. Connect the extension lines for glucose to a 3-5-fold three-way valve bench that is fixated at an appropriate place of the pen.
71. Connect a spiral extension set at the outflow of the three-way valve bench.
72. Unpack the pig's catheter ends and connect the venous catheter to a three-way valve with extension line, e.g., Discofix three-way stopcock plus extension line.
73. Connect the arterial catheter to an extension line with small diameter (0.9 × 1.9 mm) and a three-way valve.

***Note:*** The three-way valve will prevent air bubbles in the tubes.

***Note:*** The extension line helps to not disturb the pig during blood collection. The steady state is easier to achieve and can only be maintained when the pig is lying down.

74. Aspirate blood from the catheters and flush both catheters with heparinized saline.
75. Fill both extension coils up to the connection site by starting the perfusion pumps and connect the extension coils (for glucose and insulin infusion) to the three-way valve with extension line (venous catheter) (Figure 4A).

***Note:*** Mark the insulin entry port for quick access in case there is a problem.

76. Start the insulin infusion with a bolus rate (8-times the calculated continuous rate) for 2.5 min.
77. Reduce insulin infusion to the continuous infusion rate of 1 m.U. insulin per kilogram body weight per minute (1 m.U./kg BW/min.) and simultaneously start the glucose infusion.

***Note:*** The insulin infusion rate depends on the insulin sensitivity of the used model and needs to be adjusted accordingly.

***Note:*** The glucose infusion rate is variable and depends not only on the body weight of the pig but also on its insulin sensitivity: start with 80 mL/hour for a 150 kg pig and increase the rate accordingly to reach the glucose level you would like to reach.

78. Collect a small blood sample every 5 min to check the blood glucose level.
  a. Use a 3-mL syringe to aspirate heparinized saline and blood from the arterial catheter (amount dependent on the volume of the extension line and the catheter but should be the same for each animal and sample).
  b. Connect a new 3-mL syringe and collect a small blood sample.
  c. Flush the extension line with saline until there is no more blood in the line.

***Note:*** Clamp glucose levels of 80 mg/dL or 90 mg/dL.

79. Measure the blood glucose level directly after collecting the blood sample with a plasma-calibrated blood glucometer, record the value and adjust the glucose infusion rate accordingly.
80. Repeat step 78 and 79 every 5 min for a total duration of 180 min.

***Note:*** With more experience, you can reduce the blood glucose checks to a frequency of every 10 min.

***Note:*** The glucose level will vary initially but will reach a steady-state over time. Use the blood samples during the steady-state only (at least minute 160 to 180) for further evaluations.

***Note:*** To reach a stable steady-state it is important that the pig is lying down for the entire procedure, but during the steady-state in any case.

**CRITICAL:** Replace an empty perfusor syringe as quickly as possible with a new one that has already been prepared beforehand. For glucose operate at least with two perfusors as interruption of glucose or insulin infusion can disrupt the formation of the steady-state and glucose syringes need to be changed more often. Try to avoid a syringe change during the steady-state.

81. During the steady state, take a blood sample for further evaluation every 10 min, additionally to the samples for the blood glucose check.
  a. Use a 3-mL syringe to aspirate heparinized saline and blood from the arterial catheter (amount dependent on the volume of the extension line and the catheter).
  b. Connect a blood collection tube and collect the amount of blood needed.
  c. Flush the extension line with heparinized saline until there is no more blood in the line.
82. Following the last blood sampling stop the insulin infusion and continue the glucose infusion for another 10 min to prevent hypoglycemia. Confirm the blood glucose level is within the reference range by taking a final blood glucose measurement.

***Note:*** Feed the animals directly after finishing the clamp procedure.

83. Take care of the catheters after finishing the HEC:
  a. Disconnect all lines.
  b. Flush the arterial and venous catheter with heparinized saline.
  c. Add catheter lock solution.
  d. Secure the catheters into the pouch at the back of the pig again.

***Optional:*** Define prerequisites for the evaluability of the HEC, e.g. maximum permissible deviation of the glucose level during the steady state by ± 10%; maximum tolerable drop in glucose level below the desired value, lying animal during the steady state; maximum tolerable deviation from fasting time.

### Hyperglycemic clamp—Day 9

**Timing: 4–5 h**

An HGC is performed with blood sampling through the arterial catheter and glucose infusion through the venous catheter (Figure 4C).

***Note:*** The pig should be fasted for 12–18 h before starting the HGC.

**CRITICAL:** The fasting interval should be the same range for each animal from the same study (± one hour).

***Note:*** The length of infusion lines depends on your experimental setup but should be as short as possible so that changes of the infusion volume will reach the pig quickly.

84. Prepare enough heparinized saline:
  a. Remove one milliliter of saline from a 500-mL bottle.
  b. Add one milliliter of heparin (25,000 I.U. per mL) into a 500-mL bottle of saline (50 I.U. per mL saline).
  c. Shake gently and keep it at room temperature (approximately 20°C–23°C).
85. Prepare perfusor syringes with 40% glucose solution, connect them to an extension line and clamp them into a corresponding number of perfusors.

***Note:*** At least two perfusors are required for easy perfusor syringe change; whether more than two are needed depends on the body weight, insulin sensitivity and beta-cell function of the pigs.

86. Place the pig into a space-reduced pen or reduce its own pen by size.
87. Connect the extension lines for glucose to a 3-5 three-way valve bench that is fixated at an appropriate place of the pen.
88. Connect a spiral extension set at the outflow of the three-way valve bench.
89. Unpack the pig's catheter ends and connect the venous catheter to a three-way valve with extension line, e.g., Discofix three-way valve plus extension line.
90. Connect the arterial catheter to an extension line with small diameter (0.9 × 1.9 mm) and a three-way valve.

***Note:*** The extension line helps to not disturb the pig during blood collection.

91. Aspirate blood from the catheters and flush both catheters with heparinized saline.
92. Fill the spiral extension set up to the connection site by starting the perfusion pumps and connect the spiral extension set to the three-way valve with extension line (venous catheter).
93. Start the clamp with the injection of a glucose bolus (0.5 mL per kg BW).

***Note:*** Use extra syringes and inject the glucose solution as fast as possible through the venous catheter.

94. Immediately following the glucose bolus start glucose infusion at an initial rate of 1.5 x BW mL/h.

***Note:*** The glucose infusion rate is variable and depends not only on the body weight of the pig but also on its beta-cell function and insulin sensitivity; adjust the rate until the desired glucose level is reached.

***Note:*** Clamp a supraphysiological glucose level, e.g., 300 mg/dL.

95. Collect a small blood sample every 5 min to check the blood glucose level.
  a. Use a 3 mL syringe to aspirate heparinized saline and blood from the arterial catheter (amount dependent on the volume of the extension line and the catheter but should be the same for each animal and time point).
  b. Connect a new 3 mL syringe and collect a small blood sample.
  c. Flush the extension line with saline until there is no more blood in the line.

***Note:*** With more experience, you can reduce the blood glucose checks to a frequency of every 10 min.

96. Measure the blood glucose level directly after collecting the blood sample with a plasma calibrated glucometer, record the value and adjust the glucose infusion rate accordingly.
97. Repeat step 95 and 96 every 5 min for a total duration of 90 min.

***Optional:*** For a maximum beta-cell stimulation an arginine bolus (5 g) can be injected during the clamp, e.g., at time point 55 min. For that dissolve arginine powder in 20 mL of 0.9% saline and inject it through the second access of the three-way valve with extension line of the venous catheter.

***Note:*** To reach a stable glucose level it is important that the pig is lying down for the entire procedure.

**CRITICAL:** Replace an empty perfusor syringe as quickly as possible with a new one that has already been prepared. Operate at least with two perfusors.

98. Take blood samples for further evaluations every 10 min.
  a. Use a 3-mL syringe to aspirate heparinized saline and blood from the arterial catheter (amount dependent on the volume of the extension line and the catheter).
  b. Connect a blood collection tube and collect the amount of blood needed.
  c. Flush the extension line with heparinized saline until there is no more blood in the line.
99. Following the last sample drop-down glucose infusion rate to zero stepwise to avoid hypoglycemia.

***Note:*** Feed the animals directly after finishing the clamp procedure.

100. Take care of the catheters after finishing the HEC:
  a. Disconnect all lines.
  b. Flush the arterial and venous catheter with heparinized saline.
  c. Add catheter lock solution.
  d. Secure the catheters into the pouch at the back of the pig again.

***Optional:*** Define prerequisites for the evaluability of the HGC, e.g., maximum tolerable drop in glucose level below the desired value; maximum tolerable deviation from fasting time.

### Blood processing—Day 4 or 7 or 9

**Timing: 1–3 h**

The blood taken during the GTT or glucose clamps is centrifuged, serum or plasma separated and immediately frozen.

***Note:*** Start blood processing parallel to the GTT or glucose clamp.

***Note:*** The duration of this step depends on the number of samples, the size of the centrifuge and the number of assistants at the GTT or glucose clamp. Centrifuge all blood samples immediately if possible but at least 30 min after collection.

101. Immediately after blood collection, place all blood tubes – except for serum tubes – on wet ice.

***Optional:*** Use blood tubes with glucose stabilizers, e.g., S-Monovette GlucoEXACT FC, for a more stable glucose concentration. Store them at room temperature (approximately 20°C–23°C) no longer than indicated by the manufacturer and measure the glucose concentration from plasma.

102. Centrifuge the blood as soon as possible in a pre-cooled centrifuge at 4°C with 1500 x g for 20 min.

**CRITICAL:** Define a maximum duration the samples can stay on ice prior to centrifugation.

***Note:*** Leave the serum tubes at room temperature (approximately 20°C–23°C) for at least 20 min before centrifugation.

103. Pipette the supernatant after centrifugation into pre-labeled 1.5-mL tubes and immediately freeze them on dry ice.

***Optional:*** Record differences in the blood plasma as hemolysis and lipemia and define exclusion criteria.

***Note:*** Prepare separate aliquots for every planned measurement (e.g., glucose, insulin, C-peptide, glucagon).

104. Store the tubes in a freezer at −80°C. Make sure to use tightly closing tubes, to avoid sample evaporation.

**CRITICAL:** Minimize the storage duration of whole blood samples at room temperature (approximately 20°C–23°C) or even cooled, since blood cells continue to metabolize glucose.

**Pause Point:** The samples can be stored at −80°C. Analysis within a 2 months period is recommended.

### Sample analysis of glucose—Day 1 after sample collection

**Timing: 2–3 h (dependent on the number of samples to be measured at the same time)**

Blood glucose levels should be measured from plasma (tubes containing heparin or EDTA).

***Note:*** You can measure glucose values during the GTT or glucose clamp with a plasma calibrated glucometer from full blood as indicated (step 60, 80, 97). For more precise measurements, we recommend the following steps using a fully automatic analyzer.

105. Let the samples thaw at room temperature (approximately 20°C–23°C).
106. Vortex the samples and then centrifuge them at 4°C and maximum speed for 10 min.
107. Insert the samples into a fully automatic clinical chemistry analyzer and measure the samples.

### Sample analysis of insulin (ELISA)—Day 2 after sample collection

**Timing: 1 day per ELISA, e.g., Mercodia porcine insulin ELISA**

The blood insulin level can be measured from plasma (tubes containing heparin, citrate or EDTA) or serum. If you use a different assay, check the respective manual.

***Note:*** The duration of the analysis depends on the number of ELISA plates and thus on the number of samples you would like to analyze.

***Optional:*** As an alternative to the ELISA, you can also use a radioimmunoassay, e.g., Millipore Porcine Insulin RIA. Use plasma or serum and follow the manufacturer's instructions (file:///C:/Users/u8441by/Downloads/protocol-for-ifu-pi-12k-pi-12k.pdf).

108. Let the samples thaw on wet ice.
109. Vortex the samples and then centrifuge them at 4°C and maximum speed for 10 min.
110. Follow the manufacturer’s instructions dependent on the insulin ELISA you plan to use (e.g., https://www.mercodia.com/app/uploads/2022/11/10-1200-01-DfU-v-10.0.pdf).
111. Measure each sample in duplicate.

***Note:*** Use a suitable ultrasensitive ELISA for low insulin values, e.g., Mercodia ultrasensitive human insulin ELISA.

### Sample analysis of glucagon (ELISA)—Day 3 after sample collection

**Timing: 1 day per ELISA, e.g., Mercodia animal glucagon ELISA**

The blood glucagon level is measured from EDTA plasma or serum. If you use a different assay, check the respective manual.

112. Let the samples thaw on wet ice.
113. Vortex the samples and then centrifuge them at 4°C and maximum speed for 10 min.
114. Follow the manufacturer’s instructions dependent on the glucagon ELISA you plan to use (e.g., https://www.mercodia.com/app/uploads/2022/11/Directions-for-Use-10-1281-01-Version-6.0-Lot.nr-31015-32318-32057-33181-33440-33984.pdf).
115. Measure the samples in duplicate.

### Sample analysis of C-peptide (ELISA)—Day 4 after sample collection

**Timing: 1 day per ELISA, e.g., Mercodia porcine C-peptide ELISA**

The blood C-peptide level is measured from EDTA plasma or serum. If you use a different assay, check the respective manual.

116. Let the samples thaw on wet ice.
117. Vortex the samples and then centrifuge them at 4°C and maximum speed for 10 min.
118. Follow the manufacturer’s instructions dependent on the C-peptide ELISA you plan to use (e.g., https://www.mercodia.com/app/uploads/2023/06/10-1256-01-DfU-v-7.0.pdf).
119. Measure the samples in duplicate.

***Optional:*** For glucose and hormone analyses measure controls with known values (low and high concentration) at each occasion / on each ELISA plate.

***Optional:*** Define the highest coefficient of variance (%) to be tolerated for duplicate measurements (e.g., 5–10%).

### Efficiency and required resources

Table 3 is intended to give an estimate of how many tests can be carried out per day and what personnel resources are required for this. However, these figures considerably depend on various factors such as (1) number and experience of the assistants, (2) age and the resulting body weight of the pigs, (3) equipment of the respective facility (e.g., hoist crane for larger animals), and (4) blood sampling protocol (number of blood vials per time point; e.g. different anti-coagulants).

**Table 3:** Efficiency and personnel resources

Procedure	Number of animals per day	Number of persons
Surgery Ear vein catheter	4–6	3 in total
Surgery Jugular vein / Carotid artery catheter	2–3	3–4 in total
IVGTT	8	7 in total 4 persons for blood sampling 2 persons for blood glucose measurements, data recording 1 person for sample processing (one vial per time-point)
HEC	2	5 in total 2 persons for blood sampling 2 persons for blood glucose measurements, data recording, perfusor syringe exchange) 1 person for sample processing
HGC	2	5 in total 2 persons for blood sampling 2 persons for blood glucose measurements, data recording, perfusor syringe exchange) 1 person for sample processing

## Expected outcomes

**Figure 5:**
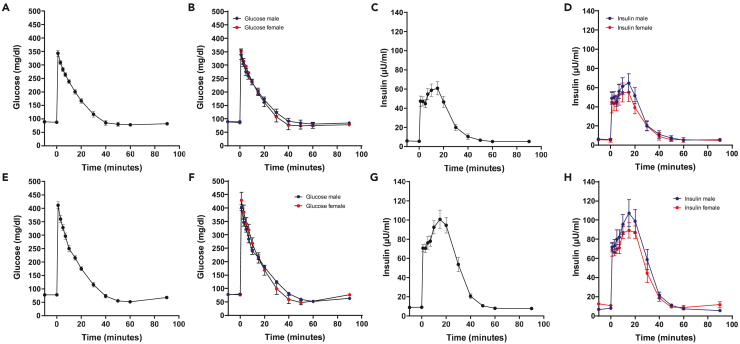
Results of an IVGTT in 3–4 month-old (A–D) and 6–7 month-old (E–H) domestic pigs Glucose concentrations following an intravenous glucose bolus of 0.5 g glucose per kg body weight in 3–4 month-old (A and B) and 6–7 month-old (E and F) domestic pigs; insulin concentrations following an intravenous glucose load of 0.5 g glucose per kg body weight in 3–4 months old (C and D) and 6–7 months old (G and H) domestic pigs. Data are means ± SEM.

**Figure 6:**
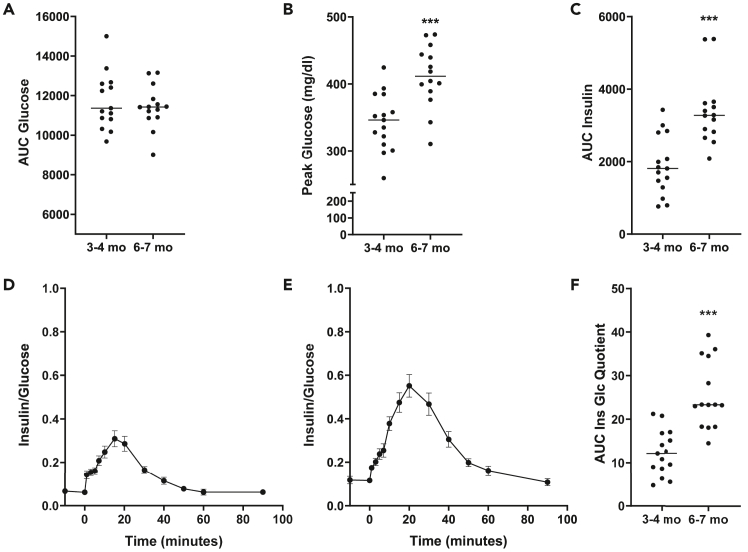
Calculations from an IVGTT in 3–4 month-old (A–D) and 6-7 month-old (E–H) domestic pigs Calculation of AUC glucose (A), Peak glucose concentrations (B), AUC insulin (C) in 3–4 months old and 6–7 months old domestic pigs. Insulin/glucose quotient in in 3–4 months old (D) and 6–7 months old (E) domestic pigs and the respective AUC of the insulin/glucose quotient (F). Data are means ± SEM. ∗∗∗: *p* < 0.001.

The intravenous glucose tolerance test (IVGTT) generally allows assessment of glucose tolerance, insulin sensitivity and beta-cell responsiveness. Especially the first phase of insulin secretion can be measured with great accuracy. At first blood samples taken prior to intravenous injection of glucose reflect basal fasting concentrations of glucose and relevant hormones for glucose metabolism like insulin, C-peptide and glucagon. Glucose is injected intravenously as a bolus, i.e., injection as fast as possible with similar injection time in all weight-matched animals. Bolus injection of glucose results in an immediate and fast rise of blood glucose levels far beyond physiological levels (Figures 5A, 5E, and 8A ), with higher peak levels (Figure 6B) but similar area under the glucose curve (AUC glucose, *p* = 0.29) within the observed age period (Figure 6A). Dependent on insulin secretion and sensitivity glucose is taken up into peripheral tissues like liver, muscle and fat resulting in a more or less steep decline of the glucose curve back to basal glucose levels (Figures 5A and 5E). The liver is the major regulator of insulin access to other peripheral tissues as up to 80% of secreted insulin is extracted during first passage through the portal vein.^21^

Upon increase of blood glucose levels insulin secretion increases with peak levels at one and 15 min following glucose load (Figures 5C and 5G) representing the first and second phase of insulin release. The strong first phase secretion peak is followed by a slower and more blunted rise in insulin secretion. Loss of glucose-induced first phase insulin secretion occurs during progression to type 1 and type 2 diabetes.^22^ However, in animals with increased body weight (from a body weight of ∼100 kg), the first peak is more difficult to depict probably also due to longer glucose injection times. Consequently, for accurate detection of an impaired insulin response, it is important to use catheters with a lumen as large as possible in animals with increased body weight. The area under the insulin curve (AUC insulin) significantly increases with age most probably related to a reduction of insulin sensitivity^23^ (Figure 6C). No gender effect can be observed in glucose and insulin concentrations during IVGTT (Figures 5B, 5D, 5F, 5H) in domestic pigs within the observed age range. Under healthy condition, glucose concentrations reach basal levels approximately 40 min following glucose load and even fall below those afterward before they return to basal levels again (Figures 5A and 5E).

The insulin–glucose quotient during IVGTT^1^ is a good measuring parameter for the evaluation of insulin sensitivity and beta-cell function as it provides information on the amount of insulin in relation to glucose disposal (Figures 6D and 6E; Table 4). Analogous to AUC insulin, AUC insulin/glucose significantly increases with age (Figure 6F).

**Table 4:** Selected examples for the interpretation of insulin-glucose quotient

Alteration in beta-cell responsiveness – insulin sensitivity	Alteration in insulin-glucose quotient
Uncompensated reduced insulin sensitivity	decrease
Compensated reduction in insulin sensitivity	increase
Increase in insulin sensitivity	decrease
Reduction in beta-cell function, i.e., insulin secretion and normal insulin sensitivity	decrease

**Figure 7:**
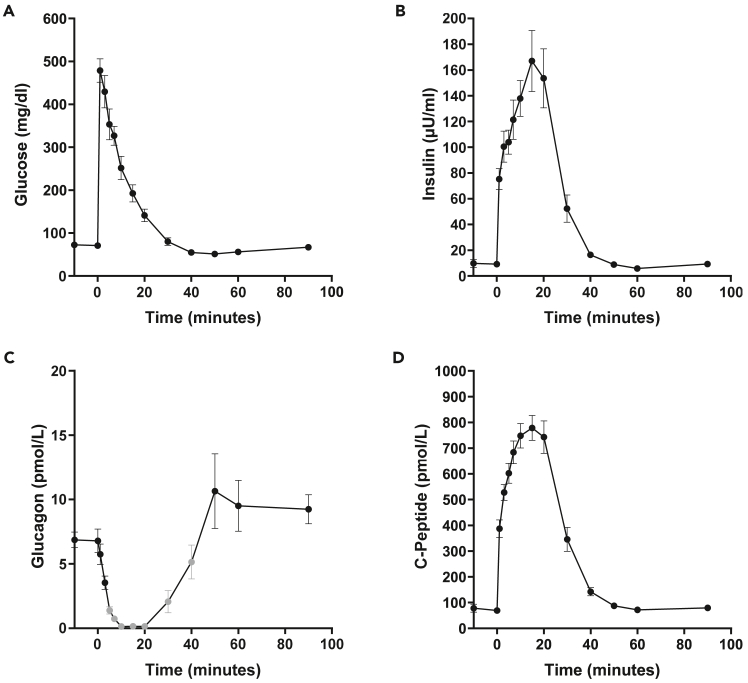
Results of an IVGTT in one-year-old minipigs Glucose (A), insulin (B), glucagon (C) and C-peptide (D) concentrations following an intravenous glucose bolus of 0.5 g glucose per kg body weight. Glucagon values below detection limit: between time point 5 min and 40 min some values had to be extrapolated (extrapolation factor 2); at time points 15 and 20 min glucagon concentrations from 2/8 animals could be detected respectively despite extrapolation. Data are means ± SEM.

In healthy animals, glucagon concentrations behave the opposite way compared to insulin concentration following an intravenous glucose load (Figure 7C).

C-peptide is secreted from pancreatic beta-cells at an equimolar ratio to insulin^24^ and in contrast to insulin is not extracted by the liver. In healthy animals, the shape of the C-peptide curve is similar to the one of the insulin curve (Figure 7D). In general, C-peptide can be used for interpretation when the evaluation of endogenous insulin secretion is difficult, e.g., in patients with exogenous insulin application.^25^ The C-peptide/insulin molar ratio can display differences in hepatic insulin fractional extraction. However, one has to consider that C-peptide and insulin have different half-lives and kinetics warranting the evaluation of additional parameters for the interpretation of alterations in glucose control.^26^

From the IVGTT different indices can be calculated to further evaluate insulin sensitivity and beta-cell function (Table 5).

**Table 5:** Calculated indices from the IVGTT

Ratio/Index	Formula	Reference
HOMA-IR	$\frac{\text{fasting}\;\text{glucose}\;\left(\text{mg}/\text{dL}\right)\;\times \;\text{fasting}\;\text{insulin}\;\left(\text{mU}/L\right)}{405}$	Insulin sensitivity^1^^,^^2^
HOMA-β	$20x\frac{\text{fasting}\;\text{insulin}\;\left(\text{mU}/L\right)}{\text{fasting}\;\text{glucose}\;\left(\text{mg}/\text{dL}\right)\text{-63}}\%$	Beta-cell function^27^
C-peptide to glucose ratio (CPRI)	$\frac{c-\text{peptide}\;\left(\text{ng}/\text{mL}\right)}{\;\text{glucose}\;\left(\text{mg}/\text{dL}\right)}\times 100$	Insulin sensitivity Beta-cell function
Quantitative insulin-sensitivity check index (QUICKI)	$\frac{1}{\log\left[\text{fasting}\;\text{insulin}\;\left(\text{mU}/L\right)\right]+\log\left[\text{fasting}\;\text{glucose}\;\left(\text{mg}/\text{dL}\right)\right]}$	Insulin sensitivity^28^
Acute insulin response to glucose (AIRg)	AUC insulin _(minutes 0–10__IVGTT)_	Beta-cell function^27^^,^^29^
Intravenous glucose tolerance index (K_G_)	Negative slope of the linear regression of ln(glucose) vs. time (5–30 min. IVGTT)	Insulin sensitivity Beta-cell function^28^
Minipig insulin sensitivity index	$\frac{30\;\times \;K_{G}}{\text{AUC}\;{\text{insulin}}_{\left(0-30\;\min.\;\text{IVGTT}\right)}}$	Insulin sensitivity^28^
C-peptide / insulin ratio	$\frac{\text{Fasting}\;c-\text{peptide}\;\left(\text{ng}/\text{mL}\right)}{\text{Fasting}\;\text{insulin}\;\left(\text{mU}/L\right)}$	Estimate of insulin clearance^30^

**Figure 8:**
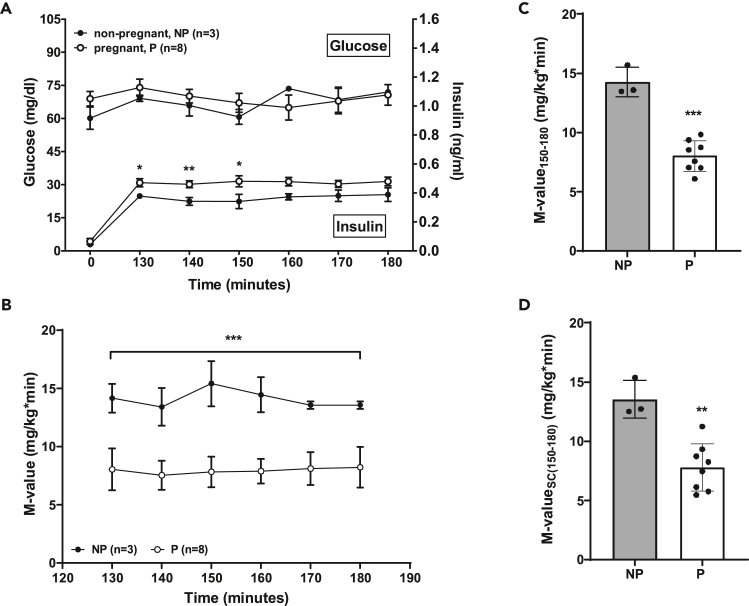
Results of a hyperinsulinemic-euglycemic clamp (HEC) in non-pregnant and pregnant domestic pigs (A) Plasma glucose and plasma insulin concentrations during the HEC (130–180 min). (B) Whole body glucose metabolism (M-value) 130–180 min. (C) Mean M-value (150–180 min). (D) Mean M-value with space correction (150–180 min). Data are means ± SEM. ∗: *p* < 0.05, ∗∗: *p* < 0.01, ∗∗∗: *p* < 0.001; adapted from Renner et al.^2^

The hyperinsulinemic-euglycemic clamp (HEC) is still the gold-standard method for the evaluation of endogenous insulin sensitivity.^31^^,^^32^^,^^33^ A continuous insulin infusion reaching supraphysiological blood insulin concentrations (Figure 8A) suppresses the hepatic glucose output and enables the investigator to evaluate peripheral glucose disposal via the glucose infusion rate as a measure of total body insulin sensitivity (Figures 8B, 8C, 8D). The primary target tissues of glucose disposal are muscle, fat and liver. The insulin infusion rate required to suppress hepatic glucose output depends on the insulin sensitivity of the animals to be evaluated. Therefore, insulin infusion rates higher than the here recommended supraphysiological rate of 1 m.U./kg BW/minute can be required. The use of a too low insulin infusion rate in the presence of hepatic insulin resistance can lead to underestimation of insulin sensitivity via sustained hepatic glucose release.

As an alternative, protocols with gradual increase of insulin infusion rates during the clamp allow the estimation of EGP suppression.^34^ However, for each insulin infusion rate a steady-state has to be reached whereby time becomes the limiting factor of so-called multi-step protocols.

To estimate hepatic glucose production stable, i.e., non-radioactively labeled glucose (e.g., deuterated glucose, 6.6-^2^H_2_-glucose) can be used.^35^^,^^36^ Infusion of stable labeled glucose for at least 120 min^35^ prior to the start of the clamp as well as during the clamp results in plasma enrichment of deuterated glucose and allows estimation of endogenous, i.e., hepatic glucose production (EGP). Also, the decline of EGP under basal and clamp condition is a measure of hepatic insulin sensitivity.

During the clamp, an euglycemic glucose level is maintained, i.e., 70–80 mg/dL for the pig (Figures 8A and S6). Over time a flow equilibrium, i.e., steady-state is established while insulin infusion rate but also the glucose infusion rate and blood glucose concentrations do not change. To maintain the steady-state, it is absolutely mandatory that the pig is lying down and not moving. Before reaching the steady-state the glucose infusion rate is variable and has to be adjusted in regular intervals. Dependent on the experience of the investigator the intervals can be shorter or longer.

Corresponding to the IVGTT, other hormones like C-peptide and glucagon can be determined during the clamp. Here, pregnant sows showed a significantly reduced glucose infusion rate (GIR) or M-value, i.e., GIR corrected for body weight, during the steady state (Figures 8B and S6) indicative of reduced insulin sensitivity during late stages of pregnancy.^2^

If EGP is fully inhibited, the glucose infusion rate (GIR) during stead-state conditions (Table 6) equals glucose disposal to peripheral tissues.^32^ To correct for minor inaccuracies in clamped glucose levels a so called space correction has been introduced by de Fronzo et al.^32^ (Table 6). If the animal pees during steady-state the M-value also needs to be corrected for urinary glucose loss (Table 6).

**Table 6:** Calculations from the HEC

Value	Formula	Reference
M-value (*body-weight-corrected glucose infusion rate, GIR*)	$\frac{\text{infused}\;\text{glucose}\;\left(\text{mg}\right)}{\text{body}\;\text{weight}\;\left(\text{kg}\right)\;\times \;\text{minute}\;\left(\min.\right)}$	Whole body insulin sensitivity^32^
Corrected M-value	GIR – Space correction (SC) – Urinary glucose loss (UC)	Whole body insulin sensitivity^32^
Space correction (SC)	$\text{SC}\;\left(\text{mg}\;\times \;{\text{kg}}^{-1}x\;\min.^{-1}\right)=\frac{\left(G2-G1\right)\;\times \;1.9\;\left(\text{dL}/\text{kg}\right)}{\left(T2-T1\right)}$	Space correction^32^
Urinary glucose loss (UC)	urinary glucose conc. (mg/dL) ∗ urinary volume (dL)	Urinary glucose loss correction^32^
M/I value	$\frac{M-\text{value}\;\left(M\right)}{\text{steady}\;\text{state}\;\text{plasma}\;\text{insulin}\;\text{conc}.\;\left(I\right)}$	M-value standardization^31^^,^^33^

G2: glucose level at the end of the steady-state; G1: glucose level at the beginning of the steady-state; T2: time at the end of the steady-state (min.); T1: time at the beginning of the steady state; 1.9 dL/kg: whole body distribution volume of glucose; conc. concentration.

**Table 7:** Calculations from the HGC

Value	Formula	Reference
M-value (*body-weight-corrected glucose infusion rate, GIR*)	$\frac{\text{infused}\;\text{glucose}\;\left(\text{mg}\right)}{\text{body}\;\text{weight}\;\left(\text{kg}\right)\;\times \;\text{minute}\;\left(\min.\right)}$	Beta-cell glucose sensitivity^37^
Corrected M-value	M-value – urinary glucose loss (UC)	Urinary glucose loss correction^37^
M/I value	$\frac{M-\text{value}\;\left(M\right)}{\text{steady}\;\text{state}\;\text{plasma}\;\text{insulin}\;\text{conc}.\;\left(I\right)}$	M-value standardization^37^
Acute insulin response to glucose (AIRg)	AUC insulin _(minutes 0–10__HGC)_	Beta-cell function^37^
Arginine stimulation	Mean Ins. _(0–10 min post inj.)_ – Mean Ins. _(0–10 min. prior to inj.)_]	Maximum insulin secretion^37^

con.: concentration; inj.: injection

**Figure 9:**
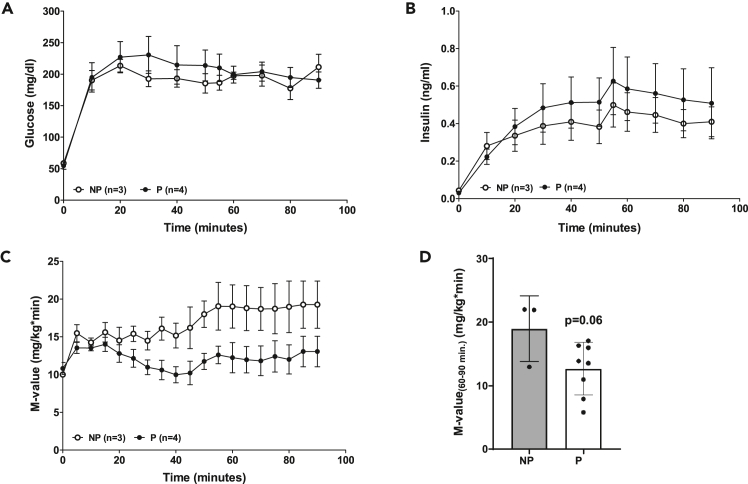
Results of a hyperglycemic clamp (HGC) in non-pregnant and pregnant domestic pigs (A) Plasma glucose and (B) plasma insulin concentrations during the HGC (0–90 min). (C) M-value (0–90 min) and (D) mean M-value (60–90 min). Data are means ± SEM.

The hyperglycemic clamp (HGC) (Figure 9) is the gold standard method for the evaluation of beta-cell function and insulin secretion and allows for the quantification of beta-cell sensitivity to glucose.^32^ In comparison to GTTs the amount of metabolized glucose can be quantified. A glucose bolus is followed by a continuous glucose infusion to reach supraphysiological levels (e.g., 200–300 mg/dL) (Figure 9A). Blood glucose levels are checked by glucometer measurements in regular, frequent intervals and the glucose infusion rate is adjusted subsequently to maintain the desired supraphysiological glucose level. To measure total insulin secretory capacity, an arginine bolus can be administered during the hyperglycemic clamp procedure (Figure 9B). Corresponding to the IVGTT, other hormones like C-peptide and glucagon can be determined during the clamp. Here, pregnant sows showed significantly reduced GIR during the HGC procedure indicative of reduced beta-cell sensitivity to glucose (Figures 9C and 9D). Related indices can also be calculated from the HGC (Table 7).

In general, indices generated from basal glucose and insulin level can also be calculated for HEC and HGC data (see Table 4).

## Quantification and statistical analysis

All data are presented as least squares means (LSMs) or means ± SEM.

Longitudinal data for glucose, insulin, C-peptide, glucagon and GIR/M-value levels as well as insulin/glucose quotient during IVGTT can be evaluated by analysis of variance (Linear Mixed Model procedure; SAS 8.2) taking the fixed effects of Group (e.g., treated vs. untreated), Time (relative to glucose administration), Gender (male/female), and the interactions (e.g., Group∗Time), as well as the random effect of animal into account.

The area under the curve and peak glucose levels can be calculated and evaluated by Mann-Whitney-U-test or Student’s t-Test respectively using e.g., GraphPad Prism software.

## Limitations

### Interpretation limitations

As glucose does not pass the digestive tract there is no dependence on variations in gastric emptying and glucose absorption from the intestine increasing the sensitivity of the IVGTT compared to an oral glucose tolerance test. However, intravenous glucose application does not induce secretion of the incretin hormones glucose-dependent insulinotropic polypeptide (GIP) and glucagon-like peptide 1 (GLP-1) from the enteroendocrine cells and does not allow any interpretation of differences in their function.

The IVGTT is a valuable tool for the detection of prediabetic and diabetic states in the pig. In humans, the oral glucose tolerance test would be the favored method due to easy oral application of glucose that can readily be done in newborn pigs by orogastric tube feeding, but is more difficult in larger, and especially ad libitum fed pigs, since these have to be trained to voluntarily take in large amounts of glucose in a short period of time. The IVGTT additionally allows interpretation on beta-cell responsiveness, secretion of other relevant hormones as e.g., glucagon, insulin sensitivity and to some degree on insulin extraction. However, the dissection of exact pathogenic mechanisms is reserved for gold standard techniques like the hyperglycemic clamp for the evaluation of beta-cell function and the hyperinsulinemic-euglycemic clamp for the evaluation of insulin sensitivity in combination with stable isotope labeling techniques.

The HEC without tracer dilution technique allows interpretation of whole-body insulin sensitivity. However, in case of hepatic insulin resistance and corresponding too low insulin infusion rate insulin sensitivity is underestimated. Correspondingly to the IVGTT, the clamp does not allow any interpretation on the incretin hormone system.

### Technical limitations

Although the placement of central venous/arterial catheters and the following GTT/clamp are good approaches for frequent blood sampling with a minimal amount of stress for the animal, there are some limitations:

The placement of the central venous catheter is limited by the number and the anatomical course of the pig’s ear veins. In long-term studies it can individually limit the study time as catheter half-life can vary due to the pig’s personality and growth rate, stable management, catheter maintenance and frequency of blood samplings. In some instances, the ear vein catheter can be replaced with a new, and longer one, and thereby allows the pig to continue in the study.

In animals with increased body weight (from a body weight of ∼100 kg), the first insulin peak is less good detectable during IVGTT. This is probably due to longer glucose injection times. To overcome this, catheters with larger lumen (4 F) can be implanted.

Jugular vein and arterial catheters equipped with suture retention beads do not allow removal without anesthesia compared to ear vein catheters.

At higher body weights, the time of glucose bolus application during the GTT increases as a larger volume of glucose solution must be given. As a result, the first time point (1 min post glucose injection) for blood sampling can no longer be sampled and must be omitted.

In case the pig does not want to lay down it is difficult to achieve a steady-state during the HEC procedure. This depends on the pig’s personality and age and can be much improved by training.

Compared to the IVGTT, the HEC and HGC are laborious, time-consuming, and more costly as well as require specifically trained and experienced personal.

## Troubleshooting

### Problem 1

Ear vein catheter: wire or catheter cannot be pushed forward during catheter placement (step 7 or 9).

### Potential solution

It can happen that the wire cannot be pushed forward into the vein for the desired length or that the central venous catheter cannot be pushed over the wire. Depending on the cause of the problem, there are several solutions.

• Move the ear in different directions and then try to push the wire or catheter forward again. In this way, the angulation of the vein can be changed and the wire or catheter can overcome possible (physiological) constrictions.
• Ask an assistant to use his or her fingers to guide the wire or catheter from the top of the ear in the required direction. To do this, apply light pressure to the skin just next to the vein and move the vein slightly back and forth. At the same time, try to push the wire or catheter further forward.
• If the wire cannot be pushed any further, slide a normal venous catheter over the wire and pull the wire out of the vein. Flush the vein with heparinized saline and then try to insert the wire into the vein again.
• If the catheter is difficult to insert into the vein through the puncture site, check the skin puncture site for its appropriate size. To do this, remove the catheter completely and guide the dilator over the wire to enlarge the insertion site.
• If the catheter cannot be pushed any further over the wire but is already an adequate distance into the vein (e.g., 15/20 cm), pull the wire out of the central venous catheter and flush the catheter with heparinized saline. While doing this, make sure that the catheter does not slip out of the ear. Insert the wire again through the central venous catheter into the vein and then try to push the catheter further forward.
• Choose another vein or use the animal’s other ear if you feel that no progress can be made in the vein.
• If no other vein is available and the catheter only protrudes a little from the vein, pull the catheter out of the vein and shorten it by the required length using a catheter guillotine to not produce any sharp edges. Slide the catheter over the wire back into the vein and check its patency by drawing blood.

### Problem 2

Ear vein, jugular vein or carotid artery catheter clotting (steps 19 and 53).

### Potential solution

Catheter clotting can occur if blood remains in the catheter or the catheter is not flushed properly. This can cause the catheter to be lost completely and endanger the continued success of the protocol.

• Preventive: Try to work as quickly and clean as possible to prevent the blood from clotting in the first place. Follow the catheter care routine and flush the catheter with sufficient heparinized saline after each blood collection. Add a catheter lock solution, e.g., Tauro Lock to the catheter every time you take a longer break (several hours).
• If there is already clotting, try to aspirate blood (esp. important for arterial catheter) and flush the catheter several times with larger amounts of heparinized saline. Use syringes with a larger volume (e.g., 10 mL or 20 mL). Try to collect blood alternating with the flushing process. When the catheter is patent again, continue with catheter care as indicated in step 19 or 53.

### Problem 3

Ear vein catheter: Catheter can be flushed but no blood can be collected (step 19 or 53).

### Potential solution

Dependent on the cause several solutions are possible.

• The catheter lumen is constricted due to the physiological position of the pig`s ear (different position compared with the position during catheter placement) or the tip of the catheter is accidentally positioned close to a venous valve:
  ○ Move the pig’s ear in different directions and ensure that the pig changes its head position to change the angulation of the vein. By changing the angle of the vein, the patency can be restored. This method is relatively easy and quick to perform and is therefore particularly suitable during a GTT.
• The adhesive tape is too loose and the catheter is bent at the insertion site:
  ○ Check the adhesive tape securing the catheter to the ear. If necessary, remove the tape to have a clear view of the catheter. If the catheter has slipped out of the ear a little and is then bent but the suture is intact, reposition it. Check the patency of the catheter by flushing and renew the adhesive tape.

### Problem 4

Ear vein, jugular vein or carotid artery catheter: Localized and/or systemic infection (steps 17 and 51).

### Potential solution

Localized infections at the catheter insertion site as well as systemic infections represent a major risk to the success of the protocol and the health of the animal. It is therefore essential to maintain the catheter as clean as possible.

• Clean the pig prior to catheter placement. Clean the pig`s ear/neck, shave and disinfect it without causing skin irritation. Implant the catheter in an operating room, use sterile material and wear sterile gloves during catheter implantation. Do not puncture a vessel when attaching the catheter to the ear. After the surgery, make sure to remove any blood residues from the puncture site (ear catheter) or wound cavity (jugular vein / carotid artery catheter). Clean the pig’s pen several times a day to keep it dry (!) and clean. Follow the regular catheter care and health check routine and wear gloves for these procedures. Do not reuse consumables (e.g., syringes, cannulas) for blood collection and flushing of the catheters.

If local inflammation occurs, treat the pig locally with an anti-inflammatory ointment. If the local inflammation does not improve or turns into local infection that is at risk for systemic infection treat the pig systemically with a suitable NSAID, antibiotic and if necessary antipyretic. Remove the catheter if there is no improvement within 2–3 days.

## Resource availability

### Lead contact

Further information and requests for resources and reagents should be directed to and will be fulfilled by the lead contact, Simone Renner (simone.renner@lmu.de).

### Technical contact

Technical questions on executing this protocol should be directed to and will be answered by the technical contact, Simone Renner (simone.renner@lmu.de).

### Materials availability

This study did not generate new unique reagents.

### Data and code availability

This study did not generate datasets or codes.

## Acknowledgments

These studies were supported by the German Center for Diabetes Research (DZD; FKZ 82DZD08D03), the EU FP7 Network for Initial Training (ITN) “EpiHealthNet” (PITN-GA-2012-317146), and—in part—the Deutsche Forschungsgemeinschaft (FOR 5795 “HyperMet”) and the EU Innovative Health Initiative “NHPig” (project 101165643).

## Author contributions

Conceptualization and study design, S.R.; methodology and investigation, Y.E., B.K., A.H., I.N., A.v.T., T.L., B.R., A.S., A.B., S.-J.K., B.Ø.C., and S.R.; writing – original draft, Y.E. and S.R.; writing – review and editing, all authors; funding acquisition, E.W., S.R., and A.H.

## Declaration of interests

The authors declare no competing interests.

## Supplemental information

Methods video S3. Implantation of a catheter into the internal jugular vein and carotid artery (steps 34–48)
