# Supplementary material for: Protocol for in vivo assessment of glucose control and insulin secretion and sensitivity in the pig
Source: STAR Protoc. 2025 Apr 16;6(2):103774. doi: 10.1016/j.xpro.2025.103774 (PMC12224880; doi:10.1016/j.xpro.2025.103774)
Supplement: Document S1. Figures S1–S6 [file mmc1.pdf]

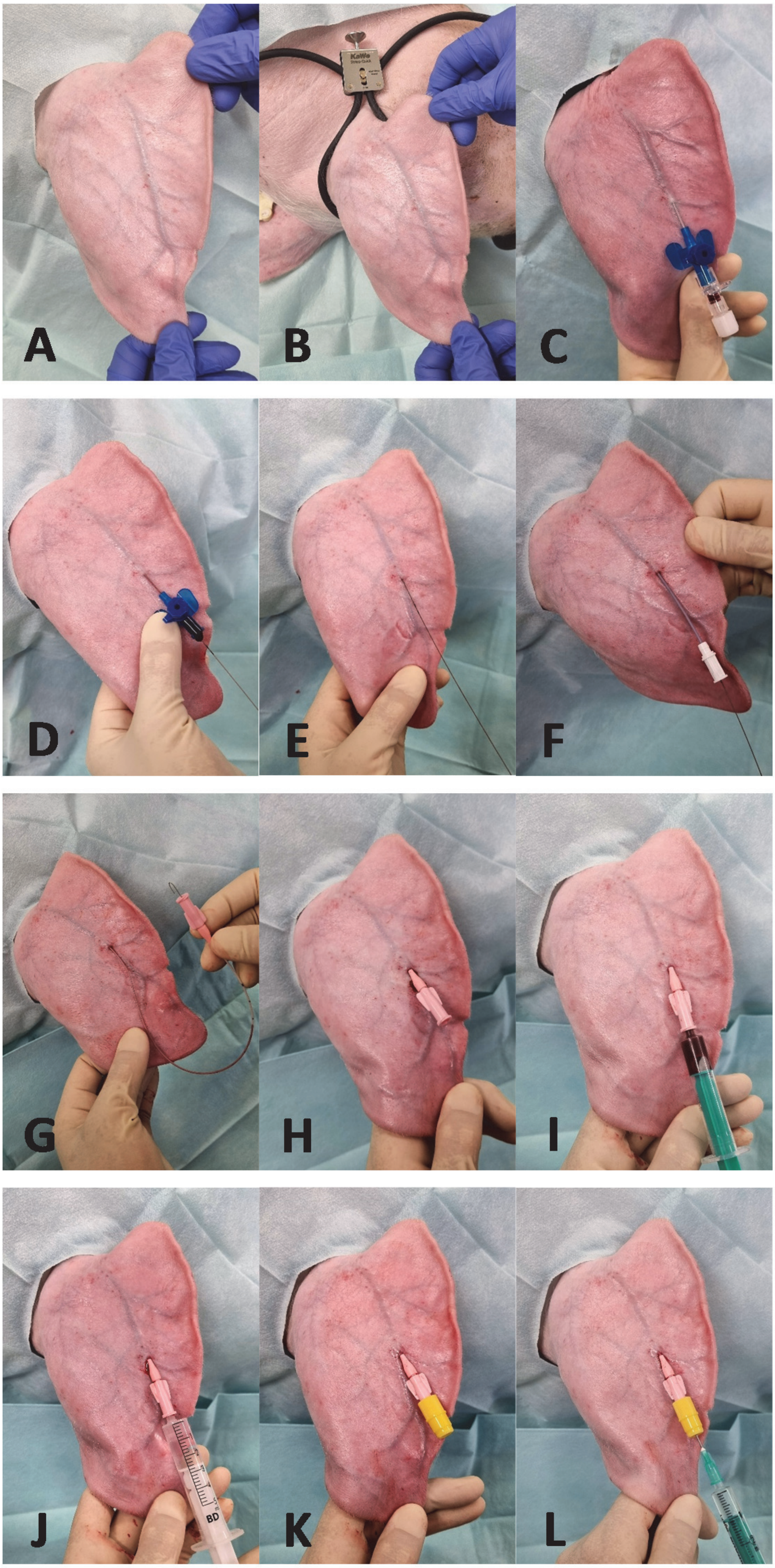

Supplemental figure 1

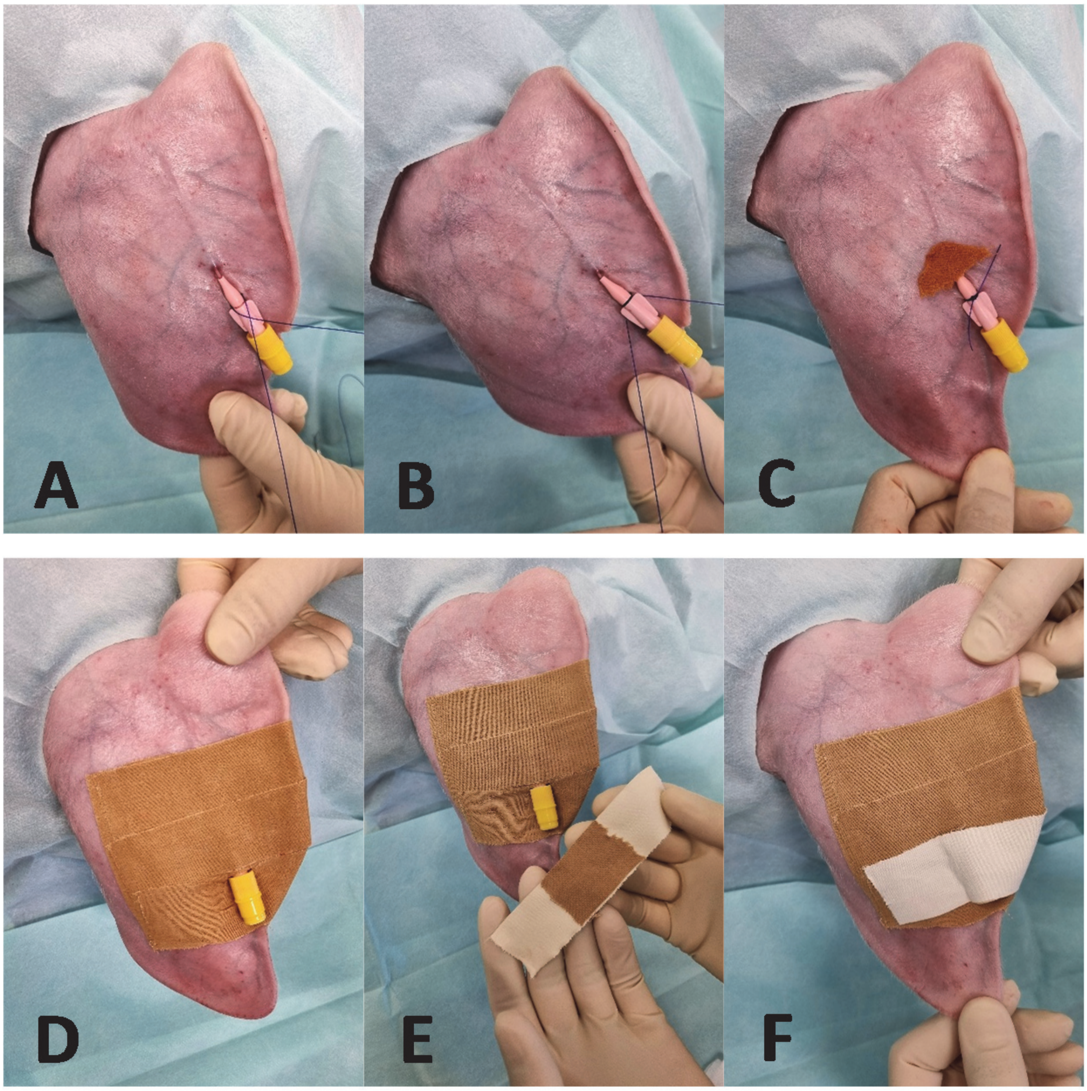

Supplemental figure 2

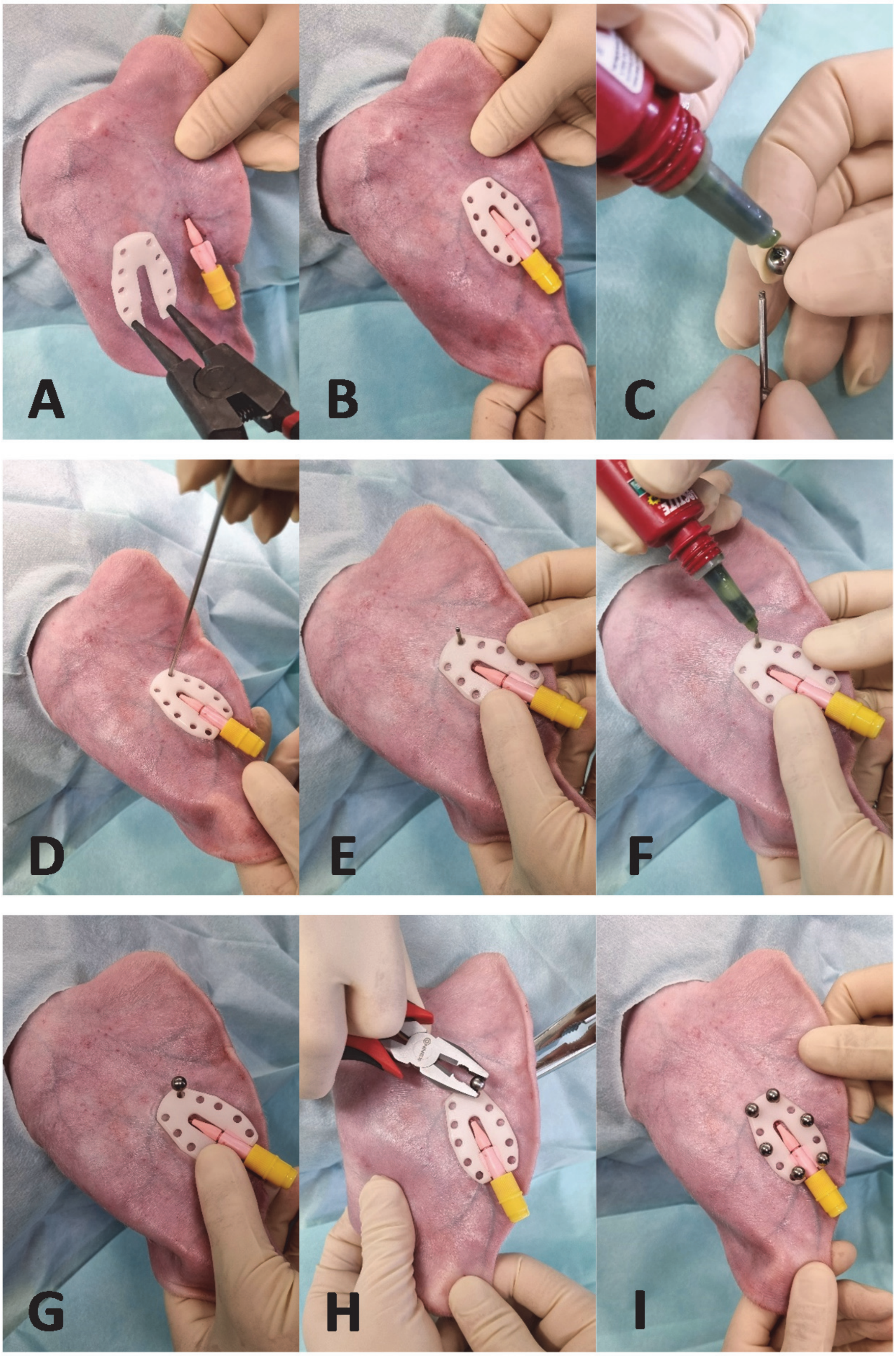

Supplemental figure 3

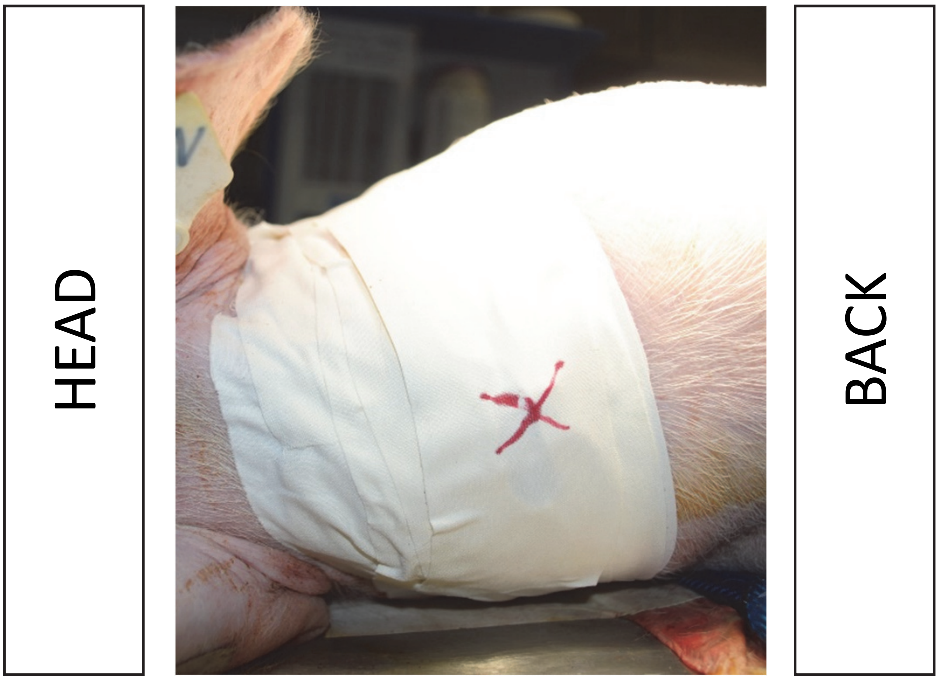

Supplemental figure 4

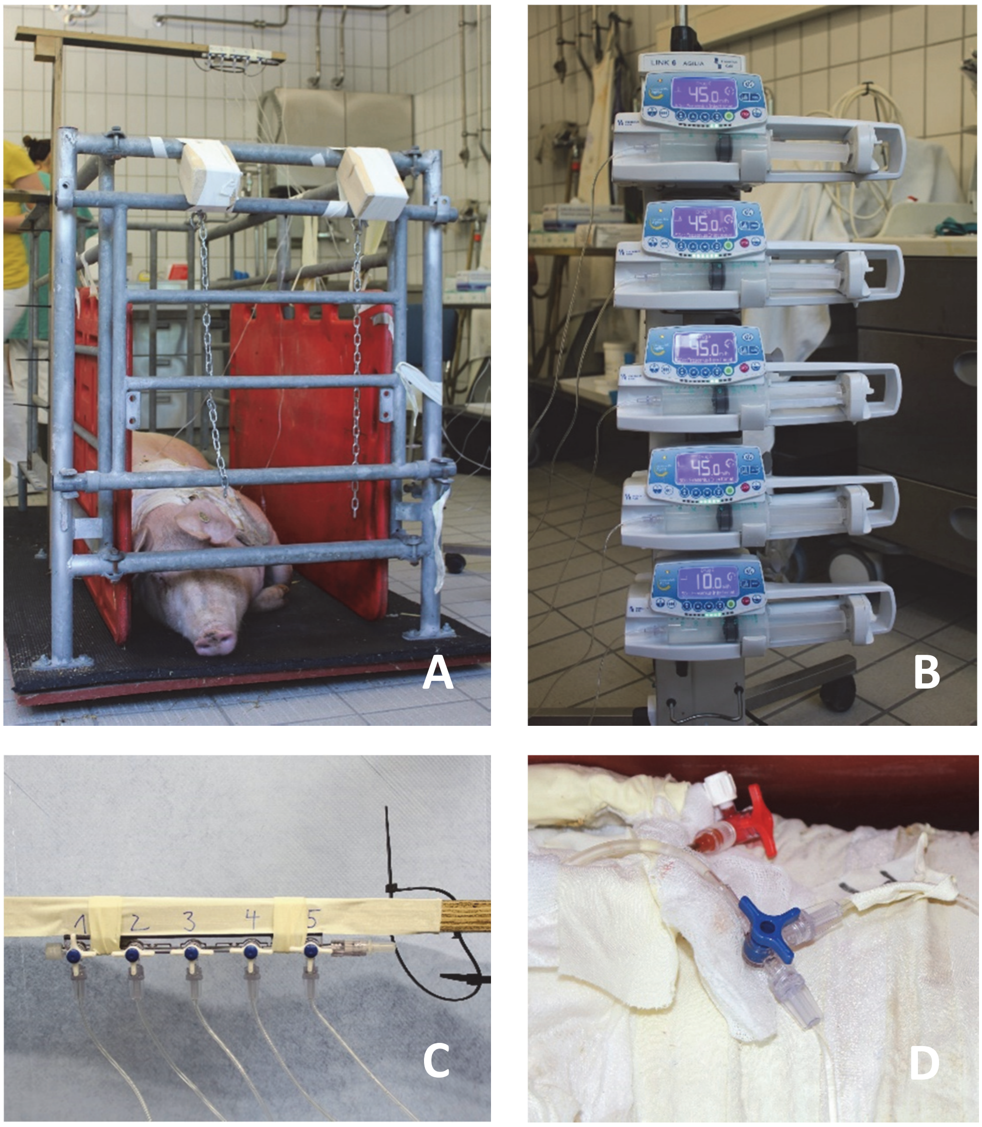

Supplemental figure 5

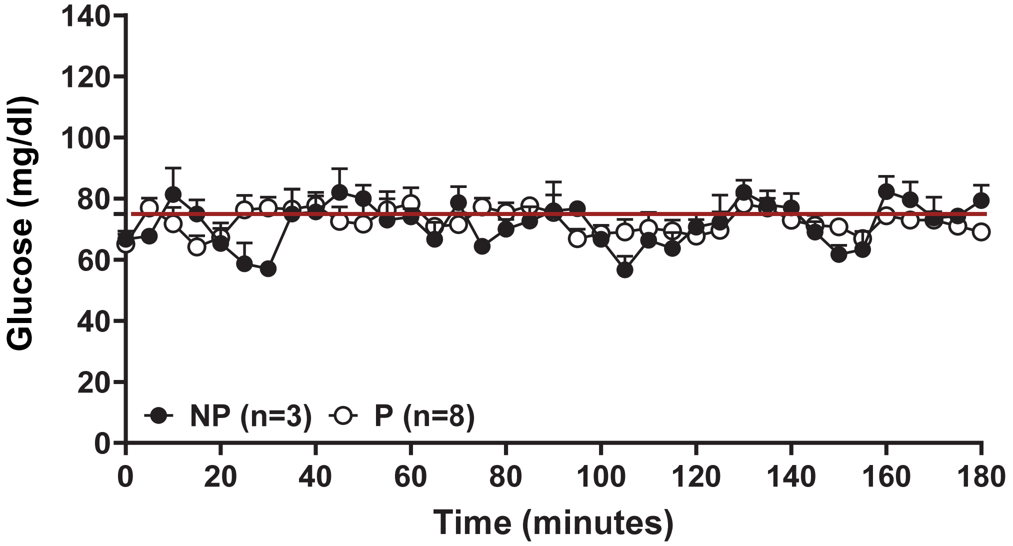

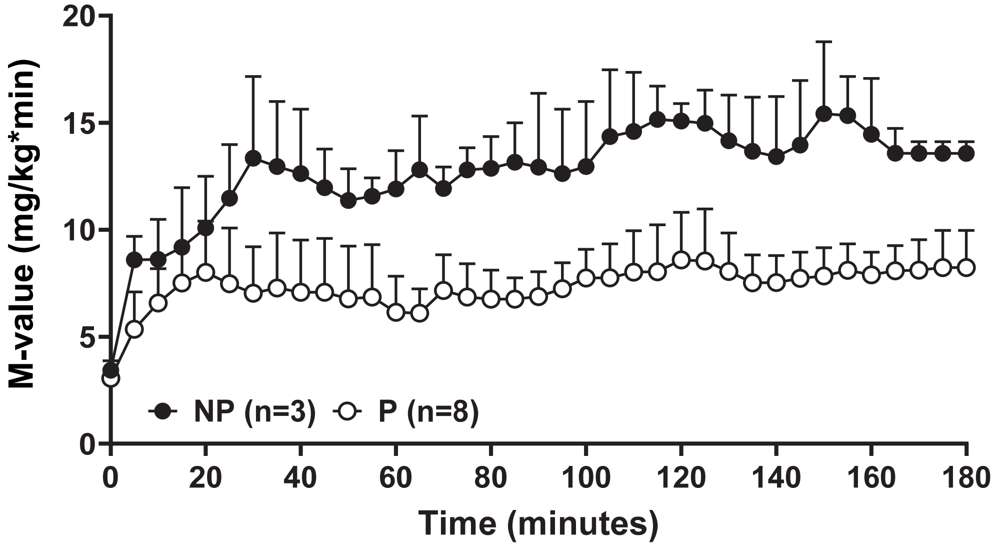

Supplemental figure 6
